# Bi-allelic variants in *DNAH3* cause male infertility with asthenoteratozoospermia in humans and mice

**DOI:** 10.1093/hropen/hoae003

**Published:** 2024-01-11

**Authors:** Gui-Quan Meng, Yaling Wang, Chen Luo, Yu-Mei Tan, Yong Li, Chen Tan, Chaofeng Tu, Qian-Jun Zhang, Liang Hu, Huan Zhang, Lan-Lan Meng, Chun-Yu Liu, Leiyu Deng, Guang-Xiu Lu, Ge Lin, Juan Du, Yue-Qiu Tan, Yanwei Sha, Lingbo Wang, Wen-Bin He

**Affiliations:** Genetic Department, Hunan Guangxiu Hospital, Hunan Normal University School of Medicine, Changsha, China; National Engineering and Research Center of Human Stem Cells & Institute of Reproductive and Stem Cell Engineering, School of Basic Medical Science, Central South University, Changsha, Hunan, China; Genetic Department, Reproductive and Genetic Hospital of CITIC-Xiangya & Clinical Research Center for Reproduction and Genetics in Hunan Province, Changsha, Hunan, China; Shanghai Key Laboratory of Metabolic Remodeling and Health, Institute of Metabolism and Integrative Biology, Institute of Reproduction and Development, Obstetrics and Gynecology Hospital, Fudan University, Shanghai, China; National Engineering and Research Center of Human Stem Cells & Institute of Reproductive and Stem Cell Engineering, School of Basic Medical Science, Central South University, Changsha, Hunan, China; Genetic Department, Reproductive and Genetic Hospital of CITIC-Xiangya & Clinical Research Center for Reproduction and Genetics in Hunan Province, Changsha, Hunan, China; GuangDong Provincial Fertility Hospital (GuangDong Provincial Reproductive Science Institute), Guangzhou, China; National Engineering and Research Center of Human Stem Cells & Institute of Reproductive and Stem Cell Engineering, School of Basic Medical Science, Central South University, Changsha, Hunan, China; Genetic Department, Reproductive and Genetic Hospital of CITIC-Xiangya & Clinical Research Center for Reproduction and Genetics in Hunan Province, Changsha, Hunan, China; National Engineering and Research Center of Human Stem Cells & Institute of Reproductive and Stem Cell Engineering, School of Basic Medical Science, Central South University, Changsha, Hunan, China; Genetic Department, Reproductive and Genetic Hospital of CITIC-Xiangya & Clinical Research Center for Reproduction and Genetics in Hunan Province, Changsha, Hunan, China; National Engineering and Research Center of Human Stem Cells & Institute of Reproductive and Stem Cell Engineering, School of Basic Medical Science, Central South University, Changsha, Hunan, China; Genetic Department, Reproductive and Genetic Hospital of CITIC-Xiangya & Clinical Research Center for Reproduction and Genetics in Hunan Province, Changsha, Hunan, China; Genetic Department, Hunan Guangxiu Hospital, Hunan Normal University School of Medicine, Changsha, China; National Engineering and Research Center of Human Stem Cells & Institute of Reproductive and Stem Cell Engineering, School of Basic Medical Science, Central South University, Changsha, Hunan, China; Genetic Department, Reproductive and Genetic Hospital of CITIC-Xiangya & Clinical Research Center for Reproduction and Genetics in Hunan Province, Changsha, Hunan, China; National Engineering and Research Center of Human Stem Cells & Institute of Reproductive and Stem Cell Engineering, School of Basic Medical Science, Central South University, Changsha, Hunan, China; Genetic Department, Reproductive and Genetic Hospital of CITIC-Xiangya & Clinical Research Center for Reproduction and Genetics in Hunan Province, Changsha, Hunan, China; National Engineering and Research Center of Human Stem Cells & Institute of Reproductive and Stem Cell Engineering, School of Basic Medical Science, Central South University, Changsha, Hunan, China; Genetic Department, Reproductive and Genetic Hospital of CITIC-Xiangya & Clinical Research Center for Reproduction and Genetics in Hunan Province, Changsha, Hunan, China; National Engineering and Research Center of Human Stem Cells & Institute of Reproductive and Stem Cell Engineering, School of Basic Medical Science, Central South University, Changsha, Hunan, China; Genetic Department, Reproductive and Genetic Hospital of CITIC-Xiangya & Clinical Research Center for Reproduction and Genetics in Hunan Province, Changsha, Hunan, China; Obstetrics and Gynecology Hospital, State Key Laboratory of Genetic Engineering, Institute of Reproduction and Development, Fudan University, Shanghai, China; Reproductive Center of No.924 Hospital of PLA Joint Logistic Support Force, Guilin, China; Genetic Department, Hunan Guangxiu Hospital, Hunan Normal University School of Medicine, Changsha, China; National Engineering and Research Center of Human Stem Cells & Institute of Reproductive and Stem Cell Engineering, School of Basic Medical Science, Central South University, Changsha, Hunan, China; Genetic Department, Reproductive and Genetic Hospital of CITIC-Xiangya & Clinical Research Center for Reproduction and Genetics in Hunan Province, Changsha, Hunan, China; Genetic Department, Hunan Guangxiu Hospital, Hunan Normal University School of Medicine, Changsha, China; National Engineering and Research Center of Human Stem Cells & Institute of Reproductive and Stem Cell Engineering, School of Basic Medical Science, Central South University, Changsha, Hunan, China; Genetic Department, Reproductive and Genetic Hospital of CITIC-Xiangya & Clinical Research Center for Reproduction and Genetics in Hunan Province, Changsha, Hunan, China; Genetic Department, Hunan Guangxiu Hospital, Hunan Normal University School of Medicine, Changsha, China; National Engineering and Research Center of Human Stem Cells & Institute of Reproductive and Stem Cell Engineering, School of Basic Medical Science, Central South University, Changsha, Hunan, China; Genetic Department, Reproductive and Genetic Hospital of CITIC-Xiangya & Clinical Research Center for Reproduction and Genetics in Hunan Province, Changsha, Hunan, China; Genetic Department, Hunan Guangxiu Hospital, Hunan Normal University School of Medicine, Changsha, China; National Engineering and Research Center of Human Stem Cells & Institute of Reproductive and Stem Cell Engineering, School of Basic Medical Science, Central South University, Changsha, Hunan, China; Genetic Department, Reproductive and Genetic Hospital of CITIC-Xiangya & Clinical Research Center for Reproduction and Genetics in Hunan Province, Changsha, Hunan, China; Department of Andrology, Women and Children’s Hospital, School of Medicine, Xiamen University, Xiamen, China; Fujian Provincial Key Laboratory of Reproductive Health Research, School of Medicine, Xiamen University, Xiamen, China; State Key Laboratory of Molecular Vaccinology and Molecular Diagnostics, School of Public Health, Xiamen University, Xiamen, China; Shanghai Key Laboratory of Metabolic Remodeling and Health, Institute of Metabolism and Integrative Biology, Institute of Reproduction and Development, Obstetrics and Gynecology Hospital, Fudan University, Shanghai, China; Genetic Department, Hunan Guangxiu Hospital, Hunan Normal University School of Medicine, Changsha, China; National Engineering and Research Center of Human Stem Cells & Institute of Reproductive and Stem Cell Engineering, School of Basic Medical Science, Central South University, Changsha, Hunan, China; Genetic Department, Reproductive and Genetic Hospital of CITIC-Xiangya & Clinical Research Center for Reproduction and Genetics in Hunan Province, Changsha, Hunan, China

**Keywords:** asthenoteratozoospermia, male infertility, *DNAH3*, bi-allelic variants, sperm flagella

## Abstract

**STUDY QUESTION:**

Are there other pathogenic genes for asthenoteratozoospermia (AT)?

**SUMMARY ANSWER:**

*DNAH3* is a novel candidate gene for AT in humans and mice.

**WHAT IS KNOWN ALREADY:**

AT is a major cause of male infertility. Several genes underlying AT have been reported; however, the genetic aetiology remains unknown in a majority of affected men.

**STUDY DESIGN, SIZE, DURATION:**

A total of 432 patients with AT were recruited in this study. *DNAH3* mutations were identified by whole-exome sequencing (WES). *Dnah3* knockout mice were generated using the genome editing tool. The morphology and motility of sperm from *Dnah3* knockout mice were investigated. The entire study was conducted over 3 years.

**PARTICIPANTS/MATERIALS, SETTING, METHODS:**

WES was performed on 432 infertile patients with AT. In addition, two lines of *Dnah3* knockout mice were generated. Haematoxylin and eosin (H&E) staining, transmission electron microscopy (TEM), immunostaining, and computer-aided sperm analysis (CASA) were performed to investigate the morphology and motility of the spermatozoa. ICSI was used to overcome the infertility of one patient and of the *Dnah3* knockout mice.

**MAIN RESULTS AND THE ROLE OF CHANCE:**

*DNAH3* biallelic variants were identified in three patients from three unrelated families. H&E staining revealed various morphological abnormalities in the flagella of sperm from the patients, and TEM and immunostaining further showed the loss of the central pair of microtubules, a dislocated mitochondrial sheath and fibrous sheath, as well as a partial absence of the inner dynein arms. In addition, the two *Dnah3* knockout mouse lines demonstrated AT. One patient and the *Dnah3* knockout mice showed good treatment outcomes after ICSI.

**LARGE SCALE DATA:**

N/A.

**LIMITATIONS, REASONS FOR CAUTION:**

This is a preliminary report suggesting that defects in *DNAH3* can lead to asthenoteratozoospermia in humans and mice. The pathogenic mechanism needs to be further examined in a future study.

**WIDER IMPLICATIONS OF THE FINDINGS:**

Our findings show that *DNAH3* is a novel candidate gene for AT in humans and mice and provide crucial insights into the biological underpinnings of this disorder. The findings may also be beneficial for counselling affected individuals.

**STUDY FUNDING/COMPETING INTEREST(S):**

This work was supported by grants from National Natural Science Foundation of China (82201773, 82101961, 82171608, 32322017, 82071697, and 81971447), National Key Research and Development Program of China (2022YFC2702604), Scientific Research Foundation of the Health Committee of Hunan Province (B202301039323, B202301039518), Hunan Provincial Natural Science Foundation (2023JJ30716), the Medical Innovation Project of Fujian Province (2020-CXB-051), the Science and Technology Project of Fujian Province (2023D017), China Postdoctoral Science Foundation (2022M711119), and Guilin technology project for people’s benefit (20180106-4-7). The authors declare no competing interests.

WHAT DOES THIS MEAN FOR PATIENTS? Asthenoteratozoospermia (AT) is a prevalent condition which affects the motility and morphology of spermatozoa. It is an underlying cause of male infertility and is usually attributed to genetic anomalies. Our study identified genetic variants in the *DNAH3* gene in three patients with AT from three unrelated families. Two lines of mice with the *DNAH3* gene knocked out demonstrated sperm characteristics similar to those in human patients. These findings indicate that *DNAH3* is a novel candidate pathogenic gene for AT and enriches the genetic aetiology of this kind of disease.

Additionally, one patient, as well as the mice with the *DNAH3* defects, showed good treatment outcomes with intracytoplasmic sperm injection (ICSI). This suggests that *DNAH3*-associated infertile males with AT have the choice of receiving clinically effective fertility therapy. Therefore, our results could provide insights for genetic counselling of individuals diagnosed with AT.

## Introduction

Infertility has become a global health problem that affects an estimated 15% of couples (Sang *et al.*, 2023), and infertility among men accounts for approximately half of all cases (Krausz and Riera-Escamilla, 2018). Asthenoteratozoospermia (AT) is a prevalent condition underlying male infertility characterized by a proportion of spermatozoa with low progressive motility (below 32%) and a low proportion with normal morphology (below 4%) (Coutton *et al.*, 2015; Tu *et al.*, 2020b). A multitude of causes and risk factors may contribute to this disorder, including genetic causes, environmental factors, socio-demographic risk factors, and behavioural/lifestyle risk factors (Tournaye *et al.*, 2017; Agarwal *et al.*, 2021; Okonofua *et al.*, 2022). Genetic deficiency is an important causal factor (Sudhakar *et al.*, 2021), and several genes have been reported to be responsible for AT, including those in the cilia and flagellum-associated protein (CFAP) family, the coiled-coil domain-containing family, and the dynein axonemal heavy chain (DNAH) family (Sha *et al.*, 2019; Tu *et al.*, 2019; Wang *et al.*, 2019; Chen *et al.*, 2021; Zhang *et al.*, 2022; Zhuang *et al.*, 2022).

The DNAH family genes encode axonemal dynein heavy chains associated with the assembly of the inner and outer dynein arms (IDAs and ODAs) of sperm flagella. Currently, 13 members of the DNAH gene family have been reported, including *DNAH1 to DNAH3*, *DNAH5 to DNAH12*, *DNAH14*, and *DNAH17*, among which 11 genes (*DNAH1, DNAH2*, *DNAH5 to DNAH12*, and *DNAH17*) are associated with male infertility (Yagi, 2009; Oud *et al.*, 2021; Levkova *et al.*, 2022). Variants in seven genes of the DNAH family (*DNAH1 and DNAH2*, *DNAH6 to DNAH8*, *DNAH10*, and *DNAH17*) have been reported to cause AT. However, whether *DNAH3* is involved in male infertility remains unclear.

In this study, the genetic aetiology of AT was investigated. Biallelic *DNAH3* variants were identified in three individuals with AT from three independent families using whole-exome sequencing (WES). Furthermore, two lines of *Dnah3* knockout mice were generated, both of which demonstrated AT. Our findings suggest that *DNAH3* is a novel candidate gene for AT in humans and mice.

## Materials and methods

### Human subjects

A total of 432 AT-affected, infertile Chinese men were recruited from the Reproductive and Genetic Hospital of CITIC-Xiangya (Changsha, China) and the Women and Children’s Hospital of Xiamen University (Fujian, China). Every individual was idiopathic with the normal number of chromosomes (46), and they presented the XY karyotype, which excluded Y-chromosome microdeletions and other potential causes of infertility, including iatrogenic injury, genital tract infection, testicular inflammation, and drug exposure. In addition, no abnormalities in height, weight, hair distribution, mental status, testicular size, or external genitalia were found upon physical examination. A total of 219 fertile Chinese men with normal semen parameters were recruited as controls. This study was approved by the Ethics Committees of the Reproductive and Genetic Hospital of CITIC-Xiangya (LL-SC-2019-034) and the Women and Children’s Hospital of Xiamen University (KY-2019-060). All participants provided written informed consent.

### WES and *in silico* bioinformatics analysis

Genomic DNA (gDNA) was extracted from peripheral blood samples using the QIAamp DNA Blood Midi Kit (Qiagen, Hilden, Germany) according to the manufacturer’s instructions, and subsequently subjected to WES analysis using the Agilent SureSelect Human All Exon V6 Kit (Agilent Technologies, Santa Clara, CA, USA) and the Illumina HiSeq 2000 or HiSeq X-TEN platform (Illumina, San Diego, CA, USA), as described previously (Tu *et al.*, 2021; Hu *et al.*, 2023). The raw reads were aligned to the human genome assembly GRCh37/hg19 using the Burrows-Wheeler Aligner after removing adaptors (Li and Durbin, 2009). Candidate pathogenic variants were identified using methods previously described in detail (Tan *et al.*, 2019). PCR and Sanger sequencing were performed to verify variants in the probands and their family members. The primers are listed in Supplementary Table S1.

The frequency of variants was assessed using public databases, including the 1000 Genomes Project, gnomAD, and gnomAD-EAS (1000 Genomes Project Consortium *et al.*, 2015; Karczewski *et al.*, 2020). The pathogenicity of variants was predicted with MutationTaster, Polymorphism Phenotyping v2 (PolyPhen-2), and combined annotation-dependent depletion (CADD) (Adzhubei *et al.*, 2010; Kircher *et al.*, 2014; Schwarz *et al.*, 2014). Conservation analyses across species were performed by aligning the amino acid sequences of *DNAH3* proteins from various species sourced from the GenBank database. Additionally, the three-dimensional structures of both wild-type (WT) and mutant DNAH3 proteins were built using Protein Homology/Analogy Recognition Engine V 2.0 (Phyre2) (http://www.sbg.bio.ic.ac.uk/∼phyre2/html/page.cgi?id=index) and visualized with PyMol software 2.5 (https://pymol.org/2/) (Kelley *et al.*, 2015).

### Semen parameter analysis and computer-aided sperm analysis

According to the guidelines of the WHO’s laboratory manual for examining and processing human semen (fifth edition), semen samples from subjects P1, P2, and P3 in this study were collected through masturbation after 2–7 days of sexual abstinence and analysed after liquefaction at 37°C for 30 min (Cooper *et al.*, 2010). Routine semen tests were performed to analyse the semen volume and sperm concentration and motility. Sperm morphology was observed using haematoxylin and eosin (H&E) staining of at least 200 spermatozoa selected from patients to assess the percentage of morphologically abnormal spermatozoa.

Semen samples from two mouse models were collected from the cauda epididymis based on previously described methods and then diluted in 1 ml of capacitation solution for incubation at 37°C for 30 min (He *et al.*, 2020). The suspension was dropped into Sperm Counting Chambers (SAS Medical, Beijing, China), and the motility of the spermatozoa was assessed using computer-aided sperm analysis (CASA) systems (HTM-TOX IVOS version 12, Hamilton Thorne Research, Beverly, MA, USA). At least three WT and mutant mice (at least 8-week-old) were analysed in each group.

### Haematoxylin–eosin staining

Haematoxylin–eosin staining of spermatozoa and testicular and epididymal tissues was performed according to a previous study (Wang *et al.*, 2019, 2021). Briefly, semen samples from patients and mice were collected, diluted, and fixed in 4% paraformaldehyde (PFA) (Sigma-Aldrich, 158127, St Louis, MO, USA) on slides for 30 min to prepare sperm smears for subsequent experiments. Testicular and epididymal tissues from mice were fixed in picric acid, 4% PFA, or Bouin’s solution (Sigma-Aldrich, HT10132) and then embedded, sectioned, processed, and cut into tissue sections. Sperm smears were hydrated by decreasing the concentration of graded ethanol, followed by staining with haematoxylin (Servicebio, G1004, Wuhan, China) and eosin (Servicebio, G1001), or haematoxylin–eosin (Solarbio Science & Technology, G1120, Beijing, China). The slides were then dehydrated with anhydrous ethanol at increasing concentrations from 50% to 100%, treated with xylene for clearing, and sealed with neutral resin for observation and imaging. The tissue sections from mice were first treated in an oven at 65°C for 2 h and then dewaxed with turpentine oil for 20 min. The subsequent processing procedures of the tissue sections were consistent with those of the sperm smears. Brain and trachea were fixed in 4% PFA, embedded in paraffin, sectioned to a 3 μm thickness, and stained with haematoxylin–eosin as previously reported (Zhou *et al.*, 2023b).

### Western blotting

Samples were homogenized using RIPA lysis buffer (Beyotime Biotechnology, Shanghai, China) supplemented with Protease Inhibitor Cocktail (Thermo Fisher Scientific, Waltham, MA, USA, or MCE, HY-K0010, Monmouth Junction, NJ, USA). After centrifugation at 13000 g for 15 min at 4°C, the supernatant of lysates was mixed with SDS-PAGE loading buffer (NCM Biotech, WB2001, Suzhou, China) and denatured at 100°C for 10 min for subsequent experiments. Western blotting (WB) analysis was performed as in our previous study (He *et al.*, 2021) with primary antibodies: rabbit polyclonal anti-DNAH3 (Shanghai Youke Biotechnology Co. Ltd, customized, 1:300, Shanghai, China), rabbit polyclonal anti-DNAH2 (Novus Biologicals, NBP2-49506, 1:500, Littleton, CO, USA), rabbit polyclonal anti-DNAH6 (Abcam, ab122333, 1:500, Cambridge, MA, USA), rabbit polyclonal anti-DNALI1 (Abcam, HPA020046, 1:500), mouse monoclonal anti-GAPDH (Affinity Biosciences, T0004, 1:5000, Cincinnati, OH, USA), and mouse monoclonal anti-α-TUBULIN (Sigma, T8203, 1:5000 or Sigma, T6199, 1:3000, St Louis, MO, USA).

### Transmission electron microscopy

Sperm samples from human subjects and mice were obtained for transmission electron microscopy (TEM) as described previously (Tan *et al.*, 2019). Sperm samples were fixed with 2.5% glutaraldehyde and OsO_4_, treated with OsO_4_ and sucrose, and dehydrated with graded concentrations of ethanol. Subsequently, the samples were embedded in Epon812, dodecenylsuccinic anhydride, methylnadic anhydride, and dimethylaminomethyl phenol. Ultrathin sections (70–90 nm) were stained with uranyl acetate and lead citrate. Images were captured using an HT7700 Hitachi electron microscope (Hitachi, Tokyo, Japan) equipped with a MegaView III digital camera (EMSIS GmbH, Münster, Germany) or a TECNAI-10 transmission electron microscope (Philips-FEI, Amsterdam, Netherlands).

### Generation of *Dnah3*-knockout mouse models

Two lines of *Dnah3*-knockout (KO) mouse models were generated. The *Dnah3* KO1 line was generated by Shanghai Model Organisms Centre, Inc. (Shanghai, China) using CRISPR-Cas9, while the KO2 line was constructed by inducing a nonsense mutation in mouse zygotes using ISTOP technology (Wang *et al.*, 2023a). Briefly, sgRNA1 (target: 5′-ATTGTTGGAGATCCAATGGG-3′) and sgRNA2 (target: 5′-TTTCCAGTAAGTATCCACAG-3′) were designed against exons 35 and 22 of the mouse *Dnah3* gene to generate the KO1 and KO2 lines, respectively. Gene editing components were prepared and injected into mouse zygotes to obtain the F0 generation, which were mated with C57BL/6 WT mice to obtain F1 generation mice. Homozygous *Dnah3* KO mice (*Dnah3^ko1/ko1^* and *Dnah3^ko2/ko2^*) were obtained by intercrossing heterozygous mice. Founder and offspring mice were genotyped by PCR and Sanger sequencing using the primers listed in Supplementary Table S2. Adult mice aged eight weeks or older were selected for this study. All animals were quarantined by the Department of Laboratory Animals. In addition, all animal experiments were conducted in accordance with the guidelines of the Institutional Animal Care and Use Committees of Central South University and Fudan University.

### Reverse-transcription PCR (RT-PCR)

RT-PCR was conducted to confirm the mRNA expression levels of *Dnah3* in various tissues and testicular tissues from C57BL/6N mice at different ages. Total RNA was extracted from the heart, liver, spleen, lung, kidney, stomach, intestine, testis, epididymis, and brain of the mice using TRIzol reagent (Invitrogen, 15596026, Carlsbad, CA, USA) and then reverse transcribed into cDNA using the PrimeScript RT reagent Kit (Takara, RR047Q, Kyoto, Japan) or Hiscript III 1st Stand cDNA Synthesis Kit (Vazyme, R312-02, Nanjing, China) according to the manufacturer’s instructions. mRNA expression levels of *β-actin* or *Gapdh* were used as internal controls. Primers used are listed in Supplementary Table S3.

### Immunofluorescence analysis

Slides with sperm smears were washed three times with 1 × phosphate-buffered saline (PBS) for 5 min and fixed with 4% PFA for 30 min. Then, samples were permeabilized with 0.5% Triton X-100 for 10 min and blocked with 5% bovine serum albumin for 1 h following by incubation overnight at 4°C with primary antibodies: rabbit polyclonal anti-SPAG6 (Sigma, HPA038440, 1:100), rabbit polyclonal anti-TOMM20 (Proteintech, 11802-1-AP, 1:100, Rosemont, IL, USA), rabbit polyclonal anti-AKAP4 (Sigma, HPA020046, 1:100), rabbit polyclonal anti-SEPT12 (Sigma, HPA041128, 1:100), and mouse monoclonal anti-Centrin (Sigma, 04-1624, 1:100). The samples were then washed three times with PBST (0.1% Tween-20) for 10 min prior to incubation for 2 h at 37°C with secondary antibodies: Alexa Fluor 488 anti-mouse IgG (Invitrogen, A21121, 1:1000, Carlsbad, CA, USA) and Alexa Fluor 555 anti-rabbit IgG (Invitrogen, A32732, 1:1000). Slides were washed three times with PBST (0.1% Tween-20) for 10 min and then stained with 4',6-diamidino-2-phenylindole (DAPI) for 10 min to label the cell nuclei. Fluorescence images were acquired using an Olympus IX51 fluorescence microscope (Olympus, Tokyo, Japan) and analysed using VideoTesT-FISH 2.0 software (VideoTesT, St Petersburg, Russia).

### Intracytoplasmic sperm injection analysis in human and mice

ICSI for human subjects was performed at the Reproductive and Genetic Hospital of CITIC-Xiangya, as described in our earlier study (Tan *et al.*, 2022). ICSI with mouse sperm was performed as previously described (Liu *et al.*, 2021; Cong *et al.*, 2022). Briefly, cauda epididymal sperm were obtained and maintained in HTF medium (Millipore, MR-070-D, Billerica, MA, USA) (Wang *et al.*, 2023b). Two-month-old B6D2F1 WT female mice were superovulated using 7.5 IU of pregnant mare serum gonadotropin, followed by 7.5 IU of hCG 48 h later, and then oocytes were collected. Sperm were injected into oocytes, and the ICSI embryos were then cultured in KSOM+AA WITH D-GLUCOSE medium (Millipore, MR-107-D) at 37°C under 5% CO_2_.

## Results

### Identification of bi-allelic *DNAH3* variants carried in infertile men with AT

**Figure 1.**
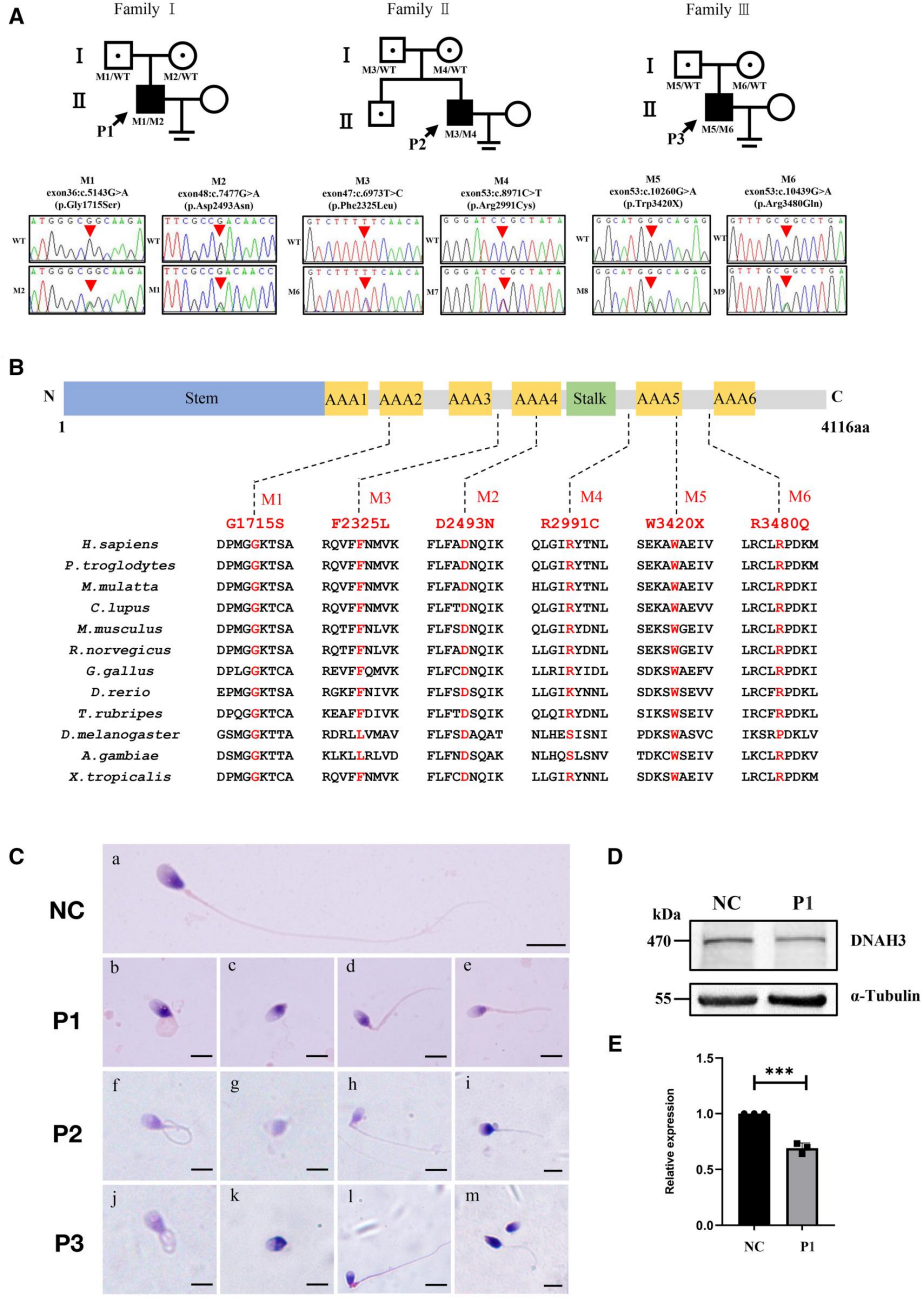
**Identification of bi-allelic *DNAH3* variants in men with asthenoteratozoospermia.** (**A**) Pedigree of three men affected by *DNAH3* variants (M1–M6) from three unrelated families. The black-filled symbol represents the infertile men harbouring bi-allelic *DNAH3* variants. Sanger sequencing chromatograms of the *DNAH3* variants (M1–M6) in all infertile males are shown at the bottom. The red arrowhead represents positions of *DNAH3* variants. (**B**) Locations and conservation analysis of *DNAH3* variants (M1–M6) identified in the patients. The human *DNAH3* gene encodes a 4116-amino acid protein that consists of an N-terminal tail, a stalk structure, and six AAA+ motor domains. (**C**) Haematoxylin–eosin (H&E) staining of the spermatozoa from the normal control (NC) and three patients with bi-allelic *DNAH3* variants. Compared with the normal morphology of the NC sperm tail (**a**), the spermatozoa of patients showed multiple morphological abnormalities of the flagella, such as coiled tail (**b**, **f**, and **j**), absent tail (**c**, **g**, and **k**), angulated tail (**d**, **h**, and **l**), and short tail (**e**, **i**, and **m**). Scale bar, 5 μm. (**D**) Western blotting of DNAH3 expression in spermatozoa from normal control (NC) and the patient (P1). α-tubulin was used as the control. (**E**) Quantification of DNAH3 levels in spermatozoa from normal control (NC) and the patient (P1). ***P < 0.001.

A cohort of 432 unrelated infertile men with AT was recruited to investigate the genetic aetiology through WES. After variant filtering and a functional analysis, ∼28% of patients had an identified genetic cause of AT, including gene mutations previously reported by our group (i.e. *DNAH10* (Tu *et al.*, 2021), *DRC3* (Zhou *et al.*, 2023a), *DNHD1* (Tan *et al.*, 2022), *SPEF2* (Tu *et al.*, 2020a), *CFAP54* (Tian *et al.*, 2023), *TTC12* (Meng *et al.*, 2023), and *CFAP65* (Wang *et al.*, 2021)), and other unreported mutations. This study focused on the novel disease-causing gene, *DNAH3*. Six variants of *DNAH3* (GenBank: NM_017539.2) were identified in three individuals (P1, P2, and P3) from three non-consanguineous families (Fig. 1A). Segregation analysis was performed via PCR-Sanger sequencing of the three families using the primers listed in Supplementary Table S1. The six mentioned variants have been submitted to the ClinVar database. In family I, the patient (P1) harboured two compound heterozygous variants c.5143G>A (p.Gly1715Ser) and c.7477G>A (p.Asp2493Asn) of *DNAH3*; in family Ⅱ, the patient (P2) also carried two compound heterozygous variants c.6973T>C (p.Phe2325Leu) and c.8971C>T (p.Arg2991Cys) of *DNAH3*; and in family III, the patient (P3) carried two compound heterozygous variants c.10260 G>A (p.Trp3420X) and c.10439G>A (p.Arg3480Gln) of *DNAH3* (Fig. 1A and Supplementary Table S4). All variants of *DNAH3* were recorded as rare or absent and predicted to be deleterious via *in silico* bioinformatics databases (1000 Genomes Project and gnomAD) and tools (PolyPhen-2, Mutation Taster, and CADD) (Supplementary Table S4). Additionally, the parents of the three patients were all heterozygous carriers in accordance with the autosomal recessive mode of inheritance (Fig. 1A).

Human *DNAH3* is predicted to encode a 4116-amino acid protein consisting of a tail, a microtubule binding domain (MTBD), and six AAA^+^ domains (Fig. 1B). These six altered amino acids were highly conserved among multiple species (Fig. 1B). Three-dimensional modelling of the DNAH3 protein (NP_060009.1) showed that the six mutations disrupted hydrogen bonds or altered the distance between atoms of adjacent amino acids, which is likely to disrupt protein stability and function (Supplementary Fig. S1). Furthermore, RT-PCR showed that *Dnah3* was abundantly expressed in the testes of mice and dramatically increased from postnatal day 21, which corresponds to the spermiogenesis stage (Supplementary Fig. S2). These findings suggest that the identified biallelic *DNAH3* variants may represent the genetic aetiology of the three AT-affected men.

### AT phenotypes in men with bi-allelic *DNAH3* variants

Routine semen analysis showed that sperm motility in three patients harbouring the bi-allelic *DNAH3* variants was dramatically decreased (Table 1). Semen samples from the three patients were collected for sperm morphology analysis using H&E staining and DNAH3 expression analysis by western blotting. Compared to spermatozoa with a normal length and smooth tail from the normal control, the spermatozoa from P1, P2, and P3 exhibited multiple morphological abnormalities of the flagella (MMAF), including short, coiled, absent, angulated, and irregular calibre flagella (Fig. 1C). In addition, the expression level of DNAH3 was lower in spermatozoa from P1 compared with that in sperm from a normal control (Fig. 1D and E). Taken together, these results demonstrate that biallelic *DNAH3* variants may contribute to the AT phenotype in infertile men.

**Table 1.** Semen characteristics and sperm morphology in the subjects harbouring *DNAH3* bi-allelic variants.

	Patient 1	Patient 2	Patient 3	Reference values
**Semen parameters**				
Semen volume (ml)	2.3	2.8	2.5	>1.5
Sperm concentration (10^6^/ml)	29.1	56.8	45.8	>15.0
Motility (%)	0	8.4	7.6	>40.0
Progressive motility (%)	0	4.6	5.8	>32.0
**Sperm flagellar morphology**				
Absent flagella (%)	23.7	23.5	17.5	<5.0
Short flagella (%)	38.8	18.4	27.5	<1.0
Coiled flagella (%)	12.1	15	11.5	<17.0
Angulation (%)	6.5	9.5	8.2	<13.0
Irregular calibre (%)	8.2	32.6	33.8	<2.0
Normal flagella (%)	10.8	1.0	1.5	>23.0

The semen parameters and sperm flagellar morphology observed in infertile individuals were evaluated according to the World Health Organization guideline (Cooper *et al.*, 2010). At least 200 spermatozoa were observed for the morphology analysis.

### *DNAH3* variants are associated with aberrant axoneme and axonemal accessory structures

**Figure 2.**
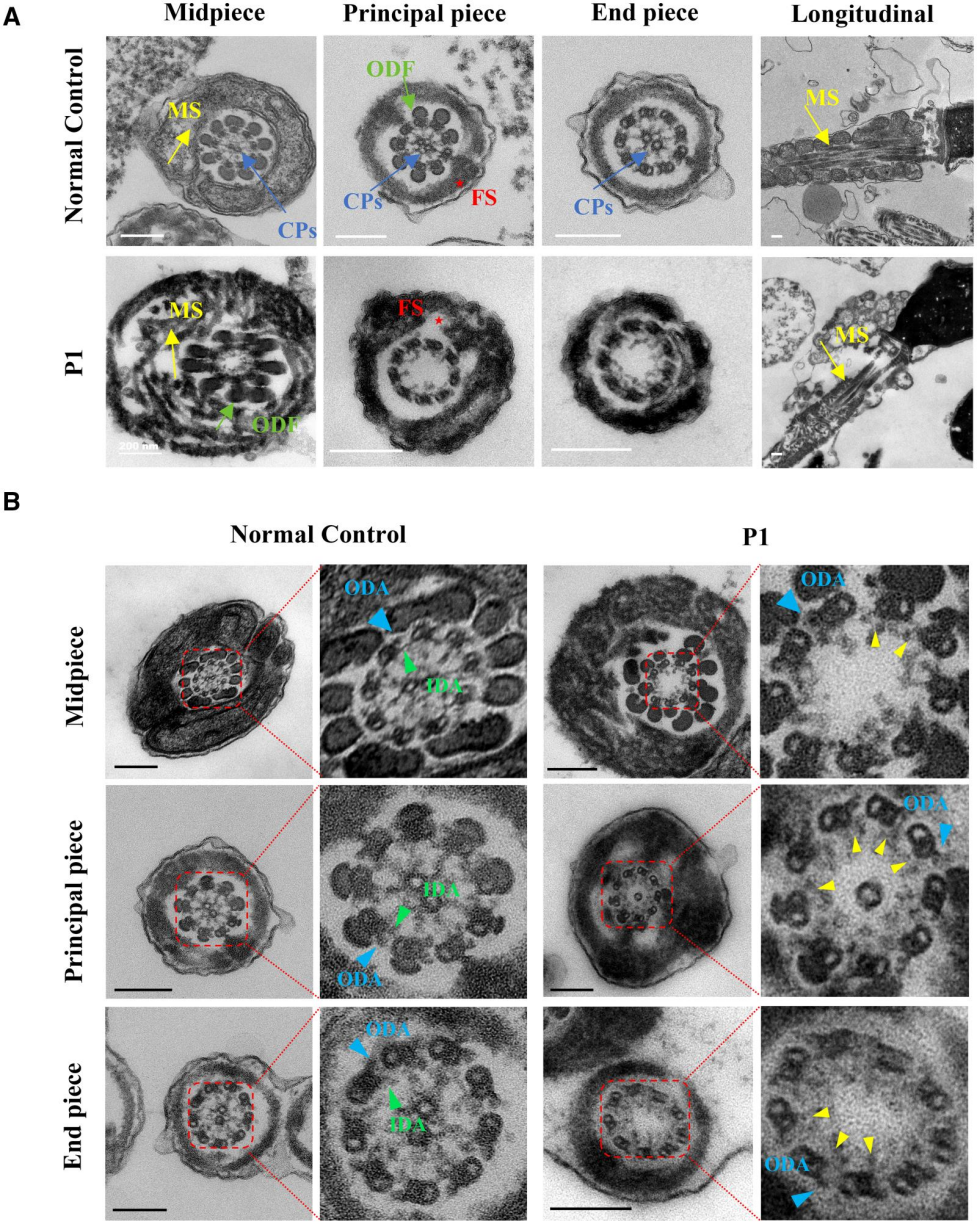
***DNAH3* variants are associated with aberrant assembly of axoneme and axonemal accessory structures.** (**A**) Transmission electron microscope (TEM) analysis of the ultrastructure of spermatozoa from normal control (NC) and the patient (P1) harbouring bi-allelic *DNAH3* variants. Cross-sections of the sperm flagella from NC showed a typical ‘9 + 2’ axoneme structure and axonemal accessory structure, including central pairs (CPs, bule arrow), outer dense fibres (ODFs, green arrow), mitochondrial sheath (MS, yellow arrow), and fibre sheath (FS, red asterisk). Cross-sections of sperm flagella from P1 revealed disordered axonemal ultrastructures, including the absence of CPs, and disorganization of MS and FS. Longitudinal sections of the sperm flagella from P1 showed poorly assembled MS and hyperplastic FS compared to the well-arranged MS and FS of sperm flagella from the NC. Scale bars, 200 nm. (**B**) TEM analysis on the axonemal dynein arms of the spermatozoa. The cross-sections of different pieces of sperm flagella from the NC showed intact inner dynein arms (IDAs; green triangle) and outer dynein arms (ODAs; blue triangle). However, the cross-sections of different pieces of sperm flagella from P1 showed partial absence of IDAs (indicated by yellow triangles). The structures in the red dotted square are magnified proportionally on the right side. Scale bars, 200 nm.

The sperm flagellum contains an important core axoneme structure, a central pair (CP) of microtubules, nine peripheral doublets of microtubules, and other accessory structures, such as the mitochondrial sheath (MS) in the mid-piece and the fibrous sheath (FS) in the principal piece. Thus, we performed a TEM analysis to observe transverse and longitudinal sections of the sperm flagella from patient P1 harbouring bi-allelic *DNAH3* variants. Compared with a normal typical “9 + 2” axoneme and accessory structure of sperm flagella from the fertile control, the sperm flagella of the patient showed obviously disordered axonemal ultrastructures, including absence of the CP and disorganization of the MS and FS (Fig. 2A). Furthermore, a poorly assembled MS was also observed in longitudinal sections of the sperm flagella (Fig. 2A). Interestingly, the IDAs were partially absent in different parts of the sperm flagella (Fig. 2B).

To confirm the ultrastructural defects revealed by TEM, immunofluorescence assays were performed to detect the expression of several proteins, including TOMM20 (component of the MS), SPAG6 (component of the CPs), and AKAP4 (component of the FS). The results showed an absent SPAG6 signal and mislocated TOMM20 and AKAP4 signals in spermatozoa from P1 (Supplementary Fig. S3A–C).

As multiple morphologically abnormal sperm flagella were displayed in men harbouring bi-allelic *DNAH3* variants, peri-axoneme structures, such as the basal body and annulus, which are involved in the formation of the sperm tail and flagellar compartmentalization, were further investigated via immunofluorescence. Spermatozoa were stained for SEPTIN12 (component of annulus) and CENTRIN (component of basal body), which showed that a bright SEPTIN12 signal was clustered at the junction between the midpiece and principal piece of normal spermatozoa. However, both the localization and expression of the SEPTIN12 signal were anomalous in spermatozoa from P1 (Supplementary Fig. S3D and E). In addition, more than two CENTRIN signals were scattered in the spermatozoa of P1, whereas two adjacent signals located at the connecting piece of normal spermatozoa (Supplementary Fig. S3F and G). The proportion of abnormal signals in the annulus and centriole in P1 was substantially higher than that in the normal controls (Supplementary Fig. S3E and G). Collectively, these results suggest that *DNAH3* is essential for the assembly of the axoneme and axonemal accessory structures in the sperm.

### *Dnah3* KO mice presented damaged male fertility with AT phenotypes

**Figure 3.**
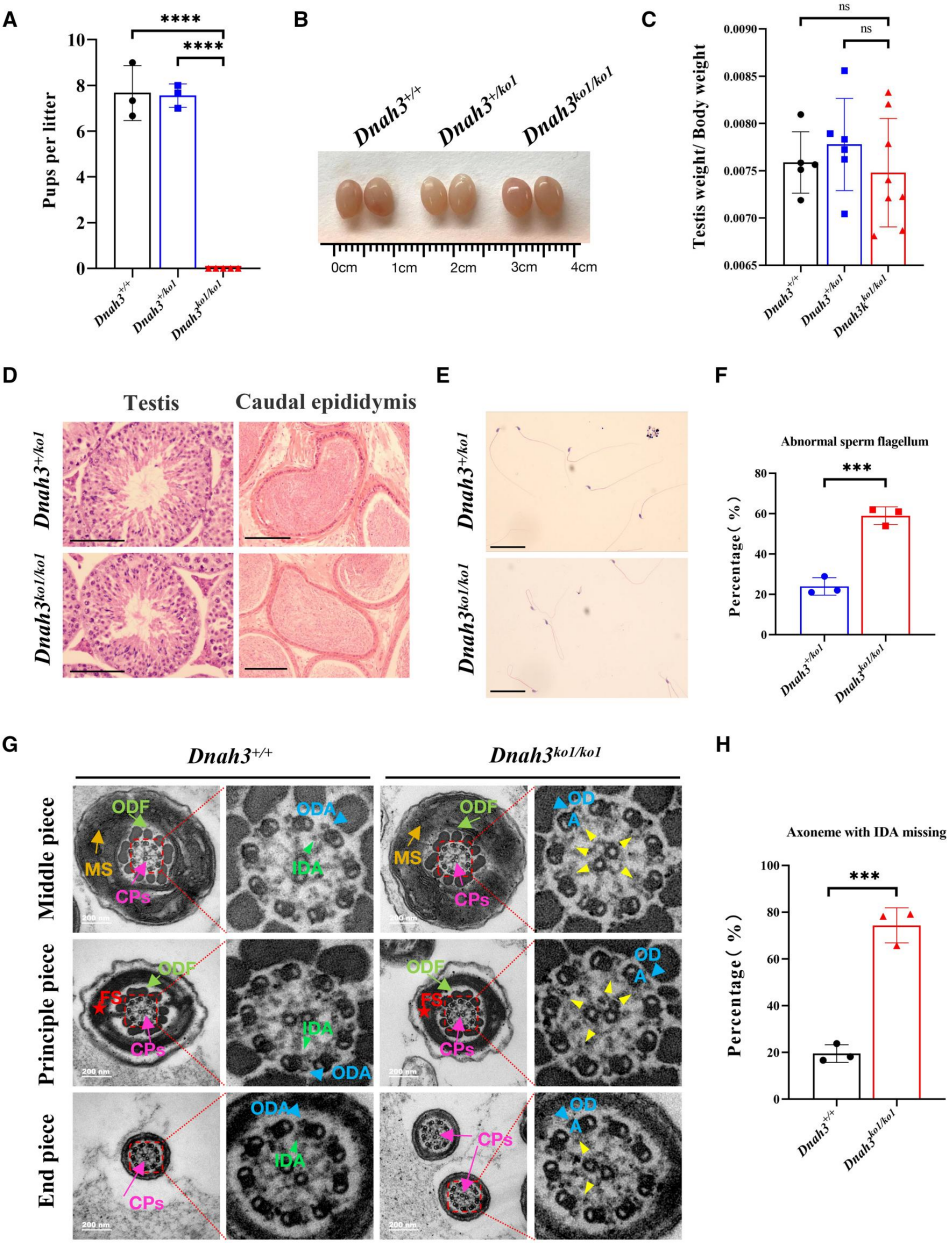
***Dnah3* deficiency replicates asthenoteratozoospermia in male mice.** (**A**) Fertility test of *Dnah3^+/+^*, *Dnah3^+/ko1^*, and *Dnah3^ko1/ko1^* male mice at 2 months of age. The *Dnah3^ko1/ko1^* male mice showed complete sterility. ****P < 0.0001. (**B**) Gross morphology comparison between the testicular size of *Dnah3^+/+^*, *Dnah3^+/ko1^*, and *Dnah3^ko1/ko1^* male mice. (**C**) Analysis of testis weight/body weight ratios between *Dnah3^+/+^*, *Dnah3^+/ko1^*, and *Dnah3^ko1/ko1^* male mice. ns indicates not significant. (**D**) H&E staining of seminiferous tubules and cauda epididymis from 2-month-old *Dnah3^+/ko1^* and *Dnah3^ko1/ko1^* male mice. Scale bars, 100 μm. (**E**) H&E staining of epididymis spermatozoa from 2-month-old *Dnah3^+/ko1^* and *Dnah3^ko1/ko1^* male mice. The aberrant curved flagellar morphology was found in spermatozoa of *Dnah3^ko1/ko1^* male mice. Scale bars, 10 μm. (**F**) Statistical data showed that the percentage of abnormal sperm flagella in *Dnah3^ko1/ko1^* male mice was significantly higher than that in *Dnah3^+/ko1^* male mice. ***P < 0.001. (**G**) Transmission electron microscopy analysis of cross-sectional ultrastructure of cauda epididymal spermatozoa from *Dnah3^+/+^* and *Dnah3^ko1/ko1^* male mice at 2 months of age. The typical ‘9 + 2’ axoneme structure and axonemal accessory structure, including central pairs (CPs; pink arrow), mitochondrial sheath (MS; yellow arrow), outer dense fibres (ODFs; green arrow), fibrous sheath (FS; red asterisk), and outer dynein arms (ODAs; blue triangle) were visible and intact in the spermatozoa from both *Dnah3^+/+^* and *Dnah3^ko1/ko1^* male mice. However, there was partial loss of inner dynein arms (indicated by yellow triangles) in the *Dnah3^ko1/ko1^* male mice compared with the intact IDAs (green triangle) in the *Dnah3^+/+^* male mice. The structures in the red dotted square are magnified proportionally on the right side. Scale bars, 200 nm. (**H**) Statistical data showing that the percentage of the axoneme with IDA missing in the sperm flagella of *Dnah3^ko1/ko1^* male mice was significantly higher than that of *Dnah3^+/+^* male mice. ***P < 0.001.

To further evaluate the role of *DNAH3* in spermatogenesis and flagellogenesis in mice, we generated two *Dnah3* KO mouse models (*Dnah3^ko1/ko1^*) and (*Dnah3^ko2/ko2^*) by inserting a base T in exon 35 of *Dnah3* (c.5039_5040 ins T) and inducing a nonsense mutation (c.3227_3228 GG>AA) in exon 22 of *Dnah3* via gene editing (Supplementary Fig. S4A and B). To further confirm the result, we analysed the potential off-target mutations in sites predicted in silico by CasOFFinder (Bae *et al.*, 2014), and observed no off-target mutations in these sites (Supplementary Table S5). Western blots revealed that DNAH3 was undetectable in the testes of *Dnah3^ko1/ko1^* and *Dnah3^ko2/ko2^* mice (Supplementary Fig. S4C and D). Fertility tests of *Dnah3^+/+^*, *Dnah3^ko1/ko1^*, and *Dnah3^ko2/ko2^* male mice (8–11 weeks old) were performed by mating these mice with fertile *Dnah3^+/+^* female mice (8 weeks old). The results showed that *Dnah3^ko1/ko1^* male mice were completely infertile, and *Dnah3^ko2/ko2^* presented severely disrupted male fertility (Fig. 3A and Supplementary Fig. S5A). Additionally, no significant differences in testicular size or testis/body weight ratio were observed between *Dnah3^+/+^* male mice and *Dnah3^ko1/ko1^* and *Dnah3^ko2/ko2^* male mice (Fig. 3B and C, Supplementary Fig. S5B and C). H&E staining revealed the presence of spermatogenic cells at different stages in the testes of *Dnah3^ko1/ko1^* and *Dnah3^ko2/ko2^* mice (Fig. 3D and Supplementary Fig. S5D).

Sperm samples were collected from the mouse cauda epididymis to analyse sperm morphology and motility. H&E staining of sperm indicated that the sperm tail of *Dnah3^ko1/ko1^* and *Dnah3^ko2/ko2^* mice showed curved and irregular morphology, with a higher proportion of abnormal sperm flagella than that of *Dnah3^+/+^* mice (Fig. 3E and F, Supplementary Fig. S5E and F). We also investigated the ultrastructure of the sperm axoneme by TEM. *Dnah3^ko1/ko1^* and *Dnah3^ko2/ko2^* male mice displayed partially absent IDAs but showed the typical “9 + 2” axonemal structure and peri-axonemal structures (Fig. 3G, Supplementary Fig. S5G). The percentage of axonemes with missing IDAs was higher in *Dnah3* knock-out mice than that in *Dnah3^+/+^* mice (Fig. 3H). Western blotting was performed to validate the IDA phenotype. The levels of DNALI1, DNAH2, and DNAH6 (different components of IDA) were lower in *Dnah3^ko1/ko1^* mice than in *Dnah3^+/+^* mice (Supplementary Fig. S6).

CASA analysis was performed to test the motility of epididymal sperm. Sperm motility, progressive motility, and swimming velocity in *Dnah3^ko1/ko1^* and *Dnah3^ko2/ko2^* mice were all lower than those in *Dnah3^+/+^* mice (Supplementary Fig. S7). Furthermore, we conducted H&E staining to explore whether cilia from trachea or brain were impaired in *Dnah3*-KO mice. We found that like WT controls, the gross ciliary morphology of trachea and brain appear normal in *Dnah3*-KO mice (Supplementary Fig. S8). Collectively, these data demonstrate that *DNAH3* defects contribute to male infertility associated with AT in both humans and mice.

### ICSI treatment on the *DNAH3*-associated male infertility

**Figure 4.**
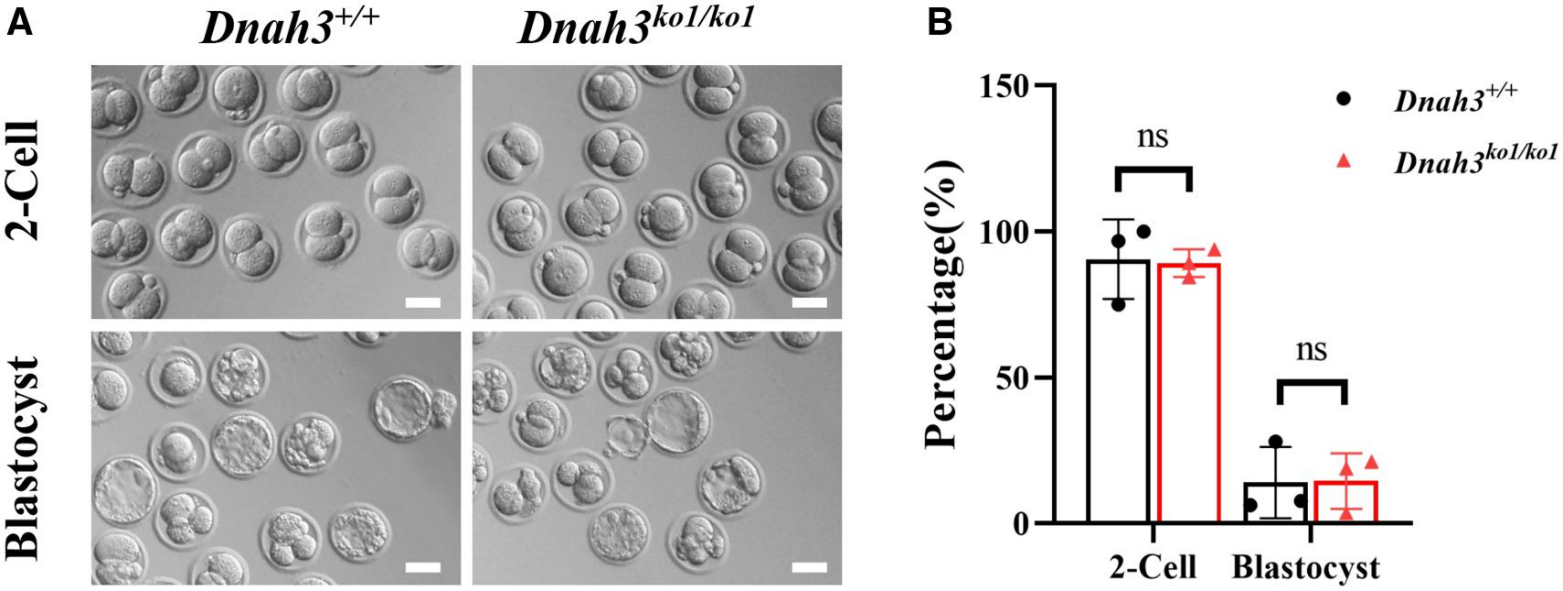
***Dnah3*-associated male infertility could be overcome by ICSI.** (**A**) Representative two-cell embryos and blastocysts of *Dnah3^+/+^* and *Dnah3^ko1/ko1^* male mice after intracytoplasmic sperm injection. Scale bar, 50 μm. (**B**) Rates of two-cell embryos and blastocysts in the *Dnah3^ko1/ko1^* group were comparable to those in the *Dnah3^+/+^* group. ns indicates not significant. n = 3 for biologically independent mice in the WT group, and n = 3 for biologically independent mice in the *Dnah3* knockout group.

To evaluate whether male infertility associated with AT caused by *DNAH3* variants can be overcome with assisted reproductive technology, ICSI was performed using the spermatozoa of *Dnah3^+/+^* and *Dnah3* KO1 male mice. The rates of two-cell embryos and blastocysts in *Dnah3* KO1 group was similar to those in the WT group (Fig. 4). Patient (P1) received ICSI treatment, and 12 embryos developed to the blastocyst stage. P1’s wife accepted a single embryo transfer and eventually became pregnant. Overall, these results indicate that ICSI may be an effective strategy for infertile men harbouring *DNAH3* variants with AT to father offspring.

## Discussion

Thirteen members of the DNAH gene family are highly expressed in human testes. Eleven of them have been found to be involved in male infertility, whereas the role of *DNAH3* and *DNAH14* in male infertility has not been clarified. In this study, we identified biallelic variants of *DNAH3* (p.Gly1715Ser and p.Asp2493Asn, p.Phe2325Leu and p.Arg2991Cys, and p.Trp3420X and p.Arg3480Gln) in three unrelated infertile men whose spermatozoa displayed multiple morphological abnormalities of the flagella and decreased motility. Subsequently, two lines of *Dnah3* KO mice were generated by inducing a frameshift or nonsense mutation, which recapitulated the patients with AT. This is the first report showing that *DNAH3* mutations are responsible for AT in humans and mice and providing new evidence for members of the DNAH gene family in spermatogenesis.

Dynein heavy chain contains eight functional domains, namely, six AAA domains with ATP hydrolysis sites, an MTBD that binds microtubules in the cleft between α- and β-tubulin, and an N-terminal tail domain that anchors to the A-microtubule of the axoneme (King, 2012; Redwine *et al.*, 2012). In our study, bi-allelic variants of *DNAH3* were identified in three infertile men and co-segregated with AT in the pedigree analysis. *DNAH3* expression was observed in several axonemal-associated structures (lung, testis, and epididymis) and restricted to the spermiogenesis stage of the testis. Additionally, variants (p.Gly1715Ser and p.Asp2493Asn) in the patient from family I (P1) were both located in the AAA domain of the *DNAH3* protein, and variants (p.Phe2325Leu, p.Arg2991Cys, and p.Arg3480Gln, respectively) in patients from families II (P2) and III (P3) were located beside the AAA domain. These five variants were shown to be highly conserved across multiple species, and the nonsense variant (p.Trp3420X) resulted only in the retention of the AAA1-AAA4 domains. Patient P1 showed the most severe phenotype with 0% sperm motility, which is a different phenotype compared to other patients with low motility. This difference could be explained by the presence of two variants (M1 and M2) in the P1 patient, both of which were located in the AAA domain. Variants M3, 4, and 6 in the other two patients (P2, P3) were not located in the AAA domain. All of the mentioned *DNAH3* variants were rare or absent in the databases and were predicted to be deleterious. Furthermore, *Dnah3* knockout mice displayed AT, and the phenotype was similar to that observed in the human patients. These results suggest that the identified novel *DNAH3* variants represent the aetiology of the three infertile males.

Dyneins are a class of motor proteins involved in intracellular cargo transport and ciliary/flagellar movement that are composed of four subunits: heavy chains, intermediate chains, light intermediate chains, and light chains (Antony *et al.*, 2021; Wu *et al.*, 2021). The DNAH family genes encode dynein axonemal heavy chains (King, 2016). Several members of the DNAH family are reportedly involved in AT (Levkova *et al.*, 2022; Yogo, 2022). Defects in these genes can induce infertility in individuals with disrupted sperm flagellar structures and severely decreased spermatozoa motility. For example, patients carrying the biallelic *DNAH17* mutation present with AT characterized by the absence of ODAs and a disorganized axoneme (Whitfield *et al.*, 2019). In our study, defects in *DNAH3* caused a partial loss of the IDAs, leading to AT in humans and mice. Our findings imply that the DNAH family genes are indispensable for the composition of sperm axonemal dynein arms.

Mouse models are useful tools for identifying novel disease genes and understanding the molecular mechanisms of human genetic diseases, because their genomes are similar to that of humans (Vandamme, 2014). However, mouse models may not completely mimic the disease state of patients in some cases, and different phenotypes between humans and mice have been described. Khan *et al.* (2021) reported that although *DNAH1* defects cause male infertility in humans and mice, the phenotypes of humans and mice present subtle differences. Male patients with *DNAH1* variants suffered from MMAF, whereas approximately half of the spermatozoa from *Dnah1* knockout mice displayed normal flagella. This may be due to the species differences between mice and humans. In our study, two lines of *Dnah3* KO mice were generated, both of which exhibited disrupted fertility. Morphological abnormalities of sperm from *Dnah3* KO mice were milder than those from individuals with defects in *DNAH3*, but the levels of several dynein proteins were decreased in *Dnah3* knockout mice. Therefore, we suggest that *DNAH3* plays a major role in the assembly of sperm flagella in humans and mice.

The mechanism underlying AT caused by the *DNAH* family genes remains largely unknown. Patients with AT resulting from a deficiency in *DNAH* family genes usually present with an aberrant distribution of axoneme and peri-axoneme structures. For example, AT-affected men harbouring biallelic variants of *DNAH1*, *DNAH2*, *DNAH6*, or *DNAH10* present with obviously absent CPs and IDAs and other structural defects in the sperm flagella (disorganized MS, outer dense fibres, and FS) (Li *et al.*, 2019, 2022; Tu *et al.*, 2019; Hu *et al.*, 2021). The mechanisms underlying this phenotype may be attributable to defects in intramanchette transport and intraflagellar transport (IFT), which are essential for sperm tail assembly (Engel *et al.*, 2012; San Agustin *et al.*, 2015; Lehti and Sironen, 2016). The proteins of the DNAH family may participate in the IFT pathway as cargo and/or motor proteins. Defective DNAH family proteins may obstruct the IFT pathway, which leads to abnormal assembly of the sperm axoneme and damage to the accessory structures (Tu *et al.*, 2021). In our study, several dynein proteins (DNAH2, DNAH6, and DNALI1) were decreased in *Dnah3* knockout mice. Additionally, the annulus of the sperm flagella was mislocalized in our patients, as has also been observed in *Dnah2* knockout male mice (Hwang *et al.*, 2021). These findings indicate that DNAH3 may participate in the assembly of sperm flagella through the IFT pathway and may play a role in the migration of the annulus in the sperm.

Although an increasing number of genes have been identified in the genetic aetiology of male infertility, there are no effective treatments to improve semen quality in infertile males with genetic defects. ICSI is an assisted reproductive technology that has been proposed as a therapeutic solution for infertile males with AT (Ferreux *et al.*, 2021). Previous studies have revealed that patients with AT caused by mutations in the *DNAH* gene family, including *DNAH1*, *DNAH2*, *DNAH7*, *DNAH8*, and *DNAH10*, can achieve pregnancy following ICSI (Wambergue *et al.*, 2016; Li *et al.*, 2019; Tu *et al.*, 2021; Wei *et al.*, 2021; Weng *et al.*, 2021). In our study, P1 carrying bi-allelic *DNAH3* variants, as well as *Dnah3* KO mice, achieved good ICSI outcomes. Therefore, our findings suggested that ICSI is beneficial for men with *DNAH3*-associated AT.

It has been reported that mutations in *DNAH3* are associated with lung, colon, and breast cancer (Tanaka *et al.*, 2008; Liu *et al.*, 2012; Hamdi *et al.*, 2018). For example, *DNAH3* is a commonly mutated gene in lung tumours, including adenocarcinomas and squamous cell carcinomas (Liu *et al.*, 2012). In addition, WES was performed on a Tunisian family with breast cancer, and the biological network construction and protein–protein interactions analyses showed that *DNAH3* might be involved in breast cancer (Hamdi *et al.*, 2018). In our study, the patients harbouring *DNAH* variants did not present with any cancers. However, we recommend that patients who carry variants in *DNAH3* or other *DNAH* family genes should undergo regular check-ups and screening for early detection, and timely intervention if cancer is identified.

The main limitation of our study is that we did not establish a mouse model for each *DNAH3* variant identified in our study. As all patients carried bi-allelic variants in *DNAH3* (one allele carries a missense variant and the other allele carries a missense variant or nonsense variant), the production of different heterozygous knock-in lines was the most appropriate method to mimic the phenotype found in humans. However, because it requires a considerable time to produce six different mutant murine lines carrying M1 to M6, we only produced two lines of KO mice. Western blotting showed that the DNAH3 protein was significant decreased in the spermatozoa from patient (P1) and was undetectable in the two lines of knock-out mice. Although there was discrepancy regarding DNAH3 protein levels between patients and KO mice, the two lines of KO mice both displayed AT, and the phenotype was similar to that observed in the human patients. Therefore, combined with the characteristics of the six variants, we suggest that *DNAH3* is a novel candidate gene for AT and that these *DNAH3* biallelic variants are the cause of infertility in the three patients.

In conclusion, *DNAH3* is a novel candidate causative gene for AT and is essential for sperm flagellar assembly and sperm motility in humans and mice. Our study provides a better understanding of the functions of *DNAH* family genes in male infertility and offers insights into the genetic aetiology and information for reproductive counselling of patients with AT.

## Supplementary Material

hoae003_Supplementary_Data

## Data Availability

The raw data supporting the conclusions of this study will be available for any qualified researcher upon reasonable requests to the corresponding author.

## References

[hoae003-B1] 1000 Genomes Project Consortium, Auton A, Brooks LD, Durbin RM, Garrison EP, Kang HM, Korbel JO, Marchini JL, McCarthy S, McVean GA et al A global reference for human genetic variation. Nature 2015;526:68–74. 26432245 10.1038/nature15393 PMC4750478

[hoae003-B2] Adzhubei IA , Schmidt S, Peshkin L, Ramensky VE, Gerasimova A, Bork P, Kondrashov AS, Sunyaev SR. A method and server for predicting damaging missense mutations. Nat Methods 2010;7:248–249. 20354512 10.1038/nmeth0410-248 PMC2855889

[hoae003-B3] Agarwal A , Baskaran S, Parekh N, Cho C-L, Henkel R, Vij S, Arafa M, Panner Selvam MK, Shah R. Male infertility. Lancet 2021;397:319–333. 33308486 10.1016/S0140-6736(20)32667-2

[hoae003-B4] Antony D , Brunner HG, Schmidts M. Ciliary dyneins and dynein related ciliopathies. Cells 2021;10:1885. 34440654 10.3390/cells10081885 PMC8391580

[hoae003-B5] Bae S , Park J, Kim J-S. Cas-OFFinder: a fast and versatile algorithm that searches for potential off-target sites of Cas9 RNA-guided endonucleases. Bioinformatics 2014;30:1473–1475. 24463181 10.1093/bioinformatics/btu048 PMC4016707

[hoae003-B6] Chen D , Liang Y, Li J, Zhang X, Zheng R, Wang X, Zhang H, Shen Y. A novel CCDC39 mutation causes multiple morphological abnormalities of the flagella in a primary ciliary dyskinesia patient. Reprod Biomed Online 2021;43:920–930. 34674941 10.1016/j.rbmo.2021.07.005

[hoae003-B7] Cong J , Yang Y, Wang X, Shen Y, Qi H-T, Liu C, Tang S, Wu S, Tian S, Zhou Y et al Deficiency of X-linked TENT5D causes male infertility by disrupting the mRNA stability during spermatogenesis. Cell Discov 2022;8:23. 35256600 10.1038/s41421-021-00369-9 PMC8901658

[hoae003-B8] Cooper TG , Noonan E, von ES, Auger J, Baker HWG, Behre HM, Haugen TB, Kruger T, Wang C, Mbizvo MT et al World Health Organization reference values for human semen characteristics. Hum Reprod Update 2010;16:231–245. 19934213 10.1093/humupd/dmp048

[hoae003-B9] Coutton C , Escoffier J, Martinez G, Arnoult C, Ray PF. Teratozoospermia: spotlight on the main genetic actors in the human. Hum Reprod Update 2015;21:455–485. 25888788 10.1093/humupd/dmv020

[hoae003-B10] Engel BD , Ishikawa H, Wemmer KA, Geimer S, Wakabayashi K, Hirono M, Craige B, Pazour GJ, Witman GB, Kamiya R et al The role of retrograde intraflagellar transport in flagellar assembly, maintenance, and function. J Cell Biol 2012;199:151–167. 23027906 10.1083/jcb.201206068 PMC3461521

[hoae003-B11] Ferreux L , Bourdon M, Chargui A, Schmitt A, Stouvenel L, Lorès P, Ray P, Lousqui J, Pocate-Cheriet K, Santulli P et al Genetic diagnosis, sperm phenotype and ICSI outcome in case of severe asthenozoospermia with multiple morphological abnormalities of the flagellum. Hum Reprod 2021;36:2848–2860. 34529793 10.1093/humrep/deab200

[hoae003-B12] Hamdi Y , Boujemaa M, Ben Rekaya M, Ben Hamda C, Mighri N, El Benna H, Mejri N, Labidi S, Daoud N, Naouali C et al; PEC Consortium. Family specific genetic predisposition to breast cancer: results from Tunisian whole exome sequenced breast cancer cases. J Transl Med 2018;16:158. 29879995 10.1186/s12967-018-1504-9 PMC5992876

[hoae003-B13] He W-B , Tan C, Zhang Y-X, Meng L-L, Gong F, Lu G-X, Lin G, Du J, Tan Y-Q. Homozygous variants in SYCP2L cause premature ovarian insufficiency. J Med Genet 2021;58:168–172. 32303603 10.1136/jmedgenet-2019-106789 PMC7907585

[hoae003-B14] He X , Liu C, Yang X, Lv M, Ni X, Li Q, Cheng H, Liu W, Tian S, Wu H et al Bi-allelic loss-of-function variants in CFAP58 cause flagellar axoneme and mitochondrial sheath defects and asthenoteratozoospermia in humans and mice. Am J Hum Genet 2020;107:514–526. 32791035 10.1016/j.ajhg.2020.07.010 PMC7477015

[hoae003-B15] Hu H-Y , Wei T-Y, Feng Z-K, Li S-J, Zhao R, Yi X-L, Hu T-L, Zhao H, Li C-X, Liu Z-G. Novel biallelic DNAH1 variations cause multiple morphological abnormalities of the sperm flagella. DNA Cell Biol 2021;40:833–840. 33989052 10.1089/dna.2021.0097

[hoae003-B16] Hu T , Meng L, Tan C, Luo C, He W-B, Tu C, Zhang H, Du J, Nie H, Lu G-X et al Biallelic CFAP61 variants cause male infertility in humans and mice with severe oligoasthenoteratozoospermia. J Med Genet 2023;60:144–153. 35387802 10.1136/jmedgenet-2021-108249

[hoae003-B17] Hwang JY , Nawaz S, Choi J, Wang H, Hussain S, Nawaz M, Lopez-Giraldez F, Jeong K, Dong W, Oh J-N et al Genetic defects in DNAH2 underlie male infertility with multiple morphological abnormalities of the sperm flagella in humans and mice. Front Cell Dev Biol 2021;9:662903. 33968937 10.3389/fcell.2021.662903 PMC8103034

[hoae003-B18] Karczewski KJ , Francioli LC, Tiao G, Cummings BB, Alföldi J, Wang Q, Collins RL, Laricchia KM, Ganna A, Birnbaum DP et al; Genome Aggregation Database Consortium. The mutational constraint spectrum quantified from variation in 141,456 humans. Nature 2020;581:434–443. 32461654 10.1038/s41586-020-2308-7 PMC7334197

[hoae003-B19] Kelley LA , Mezulis S, Yates CM, Wass MN, Sternberg MJE. The Phyre2 web portal for protein modeling, prediction and analysis. Nat Protoc 2015;10:845–858. 25950237 10.1038/nprot.2015.053 PMC5298202

[hoae003-B20] Khan R , Zaman Q, Chen J, Khan M, Ma A, Zhou J, Zhang B, Ali A, Naeem M, Zubair M et al Novel loss-of-function mutations in DNAH1 displayed different phenotypic spectrum in humans and mice. Front Endocrinol (Lausanne) 2021;12:765639. 34867808 10.3389/fendo.2021.765639 PMC8635859

[hoae003-B21] King SM. Integrated control of axonemal dynein AAA+ motors. J Struct Biol 2012;179:222–228. 22406539 10.1016/j.jsb.2012.02.013 PMC3378790

[hoae003-B22] King SM. Axonemal dynein arms. Cold Spring Harb Perspect Biol 2016;8:a028100. 27527589 10.1101/cshperspect.a028100 PMC5088525

[hoae003-B23] Kircher M , Witten DM, Jain P, O'Roak BJ, Cooper GM, Shendure J. A general framework for estimating the relative pathogenicity of human genetic variants. Nat Genet 2014;46:310–315. 24487276 10.1038/ng.2892 PMC3992975

[hoae003-B24] Krausz C , Riera-Escamilla A. Genetics of male infertility. Nat Rev Urol 2018;15:369–384. 29622783 10.1038/s41585-018-0003-3

[hoae003-B25] Lehti MS , Sironen A. Formation and function of the manchette and flagellum during spermatogenesis. Reproduction 2016;151:R43–54. 26792866 10.1530/REP-15-0310

[hoae003-B26] Levkova M , Radanova M, Angelova L. Potential role of dynein-related genes in the etiology of male infertility: a systematic review and a meta-analysis. Andrology 2022;10:1484–1499. 36057791 10.1111/andr.13287

[hoae003-B27] Li H , Durbin R. Fast and accurate short read alignment with Burrows–Wheeler transform. Bioinformatics 2009;25:1754–1760. 19451168 10.1093/bioinformatics/btp324 PMC2705234

[hoae003-B28] Li K , Wang G, Lv M, Wang J, Gao Y, Tang F, Xu C, Yang W, Yu H, Shao Z et al Bi-allelic variants in DNAH10 cause asthenoteratozoospermia and male infertility. J Assist Reprod Genet 2022;39:251–259. 34657236 10.1007/s10815-021-02306-x PMC8866613

[hoae003-B29] Li Y , Sha Y, Wang X, Ding L, Liu W, Ji Z, Mei L, Huang X, Lin S, Kong S et al DNAH2 is a novel candidate gene associated with multiple morphological abnormalities of the sperm flagella. Clin Genet 2019;95:590–600. 30811583 10.1111/cge.13525

[hoae003-B30] Liu C , Tu C, Wang L, Wu H, Houston BJ, Mastrorosa FK, Zhang W, Shen Y, Wang J, Tian S et al Deleterious variants in X-linked CFAP47 induce asthenoteratozoospermia and primary male infertility. Am J Hum Genet 2021;108:309–323. 33472045 10.1016/j.ajhg.2021.01.002 PMC7895902

[hoae003-B31] Liu P , Morrison C, Wang L, Xiong D, Vedell P, Cui P, Hua X, Ding F, Lu Y, James M et al Identification of somatic mutations in non-small cell lung carcinomas using whole-exome sequencing. Carcinogenesis 2012;33:1270–1276. 22510280 10.1093/carcin/bgs148 PMC3499051

[hoae003-B32] Meng L , Liu Q, Tan C, Xu X, He W, Hu T, Tu C, Li Y, Du J, Zhang Q et al Novel homozygous variants in TTC12 cause male infertility with asthenoteratozoospermia owing to dynein arm complex and mitochondrial sheath defects in flagella. Front Cell Dev Biol 2023;11:1184331. 37325566 10.3389/fcell.2023.1184331 PMC10267457

[hoae003-B33] Okonofua FE , Ntoimo LFC, Omonkhua A, Ayodeji O, Olafusi C, Unuabonah E, Ohenhen V. Causes and risk factors for male infertility: a scoping review of published studies. Int J Gen Med 2022;15:5985–5997. 35811778 10.2147/IJGM.S363959 PMC9268217

[hoae003-B34] Oud MS , Houston BJ, Volozonoka L, Mastrorosa FK, Holt GS, Alobaidi BKS, deVries PF, Astuti G, Ramos L, Mclachlan RI et al Exome sequencing reveals variants in known and novel candidate genes for severe sperm motility disorders. Hum Reprod 2021;36:2597–2611. 34089056 10.1093/humrep/deab099 PMC8373475

[hoae003-B35] Redwine WB , Hernandez-Lopez R, Zou S, Huang J, Reck-Peterson SL, Leschziner AE. Structural basis for microtubule binding and release by dynein. Science 2012;337:1532–1536. 22997337 10.1126/science.1224151 PMC3919166

[hoae003-B36] San Agustin JT , Pazour GJ, Witman GB. Intraflagellar transport is essential for mammalian spermiogenesis but is absent in mature sperm. Mol Biol Cell 2015;26:4358–4372. 26424803 10.1091/mbc.E15-08-0578 PMC4666132

[hoae003-B37] Sang Q , Ray PF, Wang L. Understanding the genetics of human infertility. Science 2023;380:158–163. 37053320 10.1126/science.adf7760

[hoae003-B38] Schwarz JM , Cooper DN, Schuelke M, Seelow D. MutationTaster2: mutation prediction for the deep-sequencing age. Nat Methods 2014;11:361–362. 24681721 10.1038/nmeth.2890

[hoae003-B39] Sha Y-W , Wang X, Xu X, Su Z-Y, Cui Y, Mei L-B, Huang X-J, Chen J, He X-M, Ji Z-Y et al Novel mutations in CFAP44 and CFAP43 cause multiple morphological abnormalities of the sperm flagella (MMAF). Reprod Sci 2019;26:26–34. 29277146 10.1177/1933719117749756

[hoae003-B40] Sudhakar DVS , Shah R, Gajbhiye RK. Genetics of male infertility—present and future: a narrative review. J Hum Reprod Sci 2021;14:217–227. 34759610 10.4103/jhrs.jhrs_115_21 PMC8527069

[hoae003-B41] Tan C , Meng L, Lv M, He X, Sha Y, Tang D, Tan Y, Hu T, He W, Tu C et al Bi-allelic variants in DNHD1 cause flagellar axoneme defects and asthenoteratozoospermia in humans and mice. Am J Hum Genet 2022;109:157–171. 34932939 10.1016/j.ajhg.2021.11.022 PMC8764202

[hoae003-B42] Tan Y-Q , Tu C, Meng L, Yuan S, Sjaarda C, Luo A, Du J, Li W, Gong F, Zhong C et al Loss-of-function mutations in TDRD7 lead to a rare novel syndrome combining congenital cataract and nonobstructive azoospermia in humans. Genet Med 2019;21:1209–1217. 31048812 10.1038/gim.2017.130

[hoae003-B43] Tanaka M , Jin G, Yamazaki Y, Takahara T, Takuwa M, Nakamura T. Identification of candidate cooperative genes of the Apc mutation in transformation of the colon epithelial cell by retroviral insertional mutagenesis. Cancer Sci 2008;99:979–985. 18294281 10.1111/j.1349-7006.2008.00757.x PMC11158175

[hoae003-B44] Tian S , Tu C, He X, Meng L, Wang J, Tang S, Gao Y, Liu C, Wu H, Zhou Y et al Biallelic mutations in CFAP54 cause male infertility with severe MMAF and NOA. J Med Genet 2023;60:827–834. 36593121 10.1136/jmg-2022-108887

[hoae003-B45] Tournaye H , Krausz C, Oates RD. Novel concepts in the aetiology of male reproductive impairment. Lancet Diabetes Endocrinol 2017;5:544–553. 27395771 10.1016/S2213-8587(16)30040-7

[hoae003-B46] Tu C , Cong J, Zhang Q, He X, Zheng R, Yang X, Gao Y, Wu H, Lv M, Gu Y et al Bi-allelic mutations of DNAH10 cause primary male infertility with asthenoteratozoospermia in humans and mice. Am J Hum Genet 2021;108:1466–1477. 34237282 10.1016/j.ajhg.2021.06.010 PMC8387467

[hoae003-B47] Tu C , Nie H, Meng L, Wang W, Li H, Yuan S, Cheng D, He W, Liu G, Du J et al Novel mutations in SPEF2 causing different defects between flagella and cilia bridge: the phenotypic link between MMAF and PCD. Hum Genet2020a;139:257–271. 31942643 10.1007/s00439-020-02110-0

[hoae003-B48] Tu C , Nie H, Meng L, Yuan S, He W, Luo A, Li H, Li W, Du J, Lu G et al Identification of DNAH6 mutations in infertile men with multiple morphological abnormalities of the sperm flagella. Sci Rep 2019;9:15864. 31676830 10.1038/s41598-019-52436-7 PMC6825154

[hoae003-B49] Tu C , Wang W, Hu T, Lu G, Lin G, Tan Y-Q. Genetic underpinnings of asthenozoospermia. Best Pract Res Clin Endocrinol Metab2020b;34:101472. 33191078 10.1016/j.beem.2020.101472

[hoae003-B50] Vandamme TF. Use of rodents as models of human diseases. J Pharm Bioallied Sci 2014;6:2–9. 24459397 10.4103/0975-7406.124301 PMC3895289

[hoae003-B51] Wambergue C , Zouari R, Fourati Ben Mustapha S, Martinez G, Devillard F, Hennebicq S, Satre V, Brouillet S, Halouani L, Marrakchi O et al Patients with multiple morphological abnormalities of the sperm flagella due to DNAH1 mutations have a good prognosis following intracytoplasmic sperm injection. Hum Reprod 2016;31:1164–1172. 27094479 10.1093/humrep/dew083

[hoae003-B52] Wang W , Tian S, Nie H, Tu C, Liu C, Li Y, Li D, Yang X, Meng L, Hu T et al CFAP65 is required in the acrosome biogenesis and mitochondrial sheath assembly during spermiogenesis. Hum Mol Genet 2021;30:2240–2254. 34231842 10.1093/hmg/ddab185

[hoae003-B53] Wang W , Tu C, Nie H, Meng L, Li Y, Yuan S, Zhang Q, Du J, Wang J, Gong F et al Biallelic mutations in CFAP65 lead to severe asthenoteratospermia due to acrosome hypoplasia and flagellum malformations. J Med Genet 2019;56:750–757. 31413122 10.1136/jmedgenet-2019-106031 PMC6860412

[hoae003-B54] Wang Y , Chen J, Huang X, Wu B, Dai P, Zhang F, Li J, Wang L. Gene-knockout by iSTOP enables rapid reproductive disease modeling and phenotyping in germ cells of the founder generation. Sci China Life Sci2023a;doi: 10.1007/s11427-023-2408-2. 38332217

[hoae003-B55] Wang Y , Huang X, Sun G, Chen J, Wu B, Luo J, Tang S, Dai P, Zhang F, Li J et al Coiled-coil domain-containing 38 is required for acrosome biogenesis and fibrous sheath assembly in mice. J Genet Genomics2023b;doi: 10.1016/j.jgg.2023.09.002. 10.1016/j.jgg.2023.09.002 37709195

[hoae003-B56] Wei X , Sha Y, Wei Z, Zhu X, He F, Zhang X, Liu W, Wang Y, Lu Z. Bi-allelic mutations in DNAH7 cause asthenozoospermia by impairing the integrality of axoneme structure. Acta Biochim Biophys Sin (Shanghai) 2021;53:1300–1309. 34476482 10.1093/abbs/gmab113

[hoae003-B57] Weng M , Sha Y, Zeng YU, Huang N, Liu W, Zhang X, Zhou H. Mutations in DNAH8 contribute to multiple morphological abnormalities of sperm flagella and male infertility. Acta Biochim Biophys Sin (Shanghai) 2021;53:472–480. 33704367 10.1093/abbs/gmab013

[hoae003-B58] Whitfield M , Thomas L, Bequignon E, Schmitt A, Stouvenel L, Montantin G, Tissier S, Duquesnoy P, Copin B, Chantot S et al Mutations in DNAH17, encoding a sperm-specific axonemal outer dynein arm heavy chain, cause isolated male infertility due to asthenozoospermia. Am J Hum Genet 2019;105:198–212. 31178125 10.1016/j.ajhg.2019.04.015 PMC6612517

[hoae003-B59] Wu S , Li H, Wang L, Mak N, Wu X, Ge R, Sun F, Cheng CY. Motor proteins and spermatogenesis. Adv Exp Med Biol 2021;1288:131–159. 34453735 10.1007/978-3-030-77779-1_7

[hoae003-B60] Yagi T. Bioinformatic approaches to dynein heavy chain classification. Methods Cell Biol 2009;92:1–9. 20409795 10.1016/S0091-679X(08)92001-X

[hoae003-B61] Yogo K. Molecular basis of the morphogenesis of sperm head and tail in mice. Reprod Med Biol 2022;21:e12466. 35619659 10.1002/rmb2.12466 PMC9126569

[hoae003-B62] Zhang R , Wu B, Liu C, Zhang Z, Wang X, Wang L, Xiao S, Chen Y, Wei H, Jiang H et al CCDC38 is required for sperm flagellum biogenesis and male fertility in mice. Development 2022;149:dev200516. 35587122 10.1242/dev.200516

[hoae003-B63] Zhou S , Yuan S, Zhang J, Meng L, Zhang X, Liu S, Lu G, Lin G, Liu M, Tan Y-Q. DRC3 is an assembly adapter of the nexin-dynein regulatory complex functional components during spermatogenesis in humans and mice. Signal Transduct Target Ther2023a;8:26. 36627292 10.1038/s41392-022-01293-4 PMC9832115

[hoae003-B64] Zhou Y , Wang Y, Chen J, Wu B, Tang S, Zhang F, Liu C, Wang L. Dnali1 is required for sperm motility and male fertility in mice. Basic Clin Androl2023b;33:32. 37993789 10.1186/s12610-023-00205-y PMC10666298

[hoae003-B65] Zhuang B-J , Xu S-Y, Dong L, Zhang P-H, Zhuang B-L, Huang X-P, Li G-S, You Y-D, Chen D, Yu X-J et al Novel DNAH1 mutation loci lead to multiple morphological abnormalities of the sperm flagella and literature review. World J Mens Health 2022;40:551–560. 35118838 10.5534/wjmh.210119 PMC9482856

