# Supplementary material for: Bi-allelic variants in DNAH3 cause male infertility with asthenoteratozoospermia in humans and mice
Source: Hum Reprod Open. 2024 Jan 11;2024(1):hoae003. doi: 10.1093/hropen/hoae003 (PMC10834362; doi:10.1093/hropen/hoae003)
Supplement: hoae003_Supplementary_Data [file hoae003_supplementary_data.zip › 20231221DNAH3-supplementary_figs.docx]

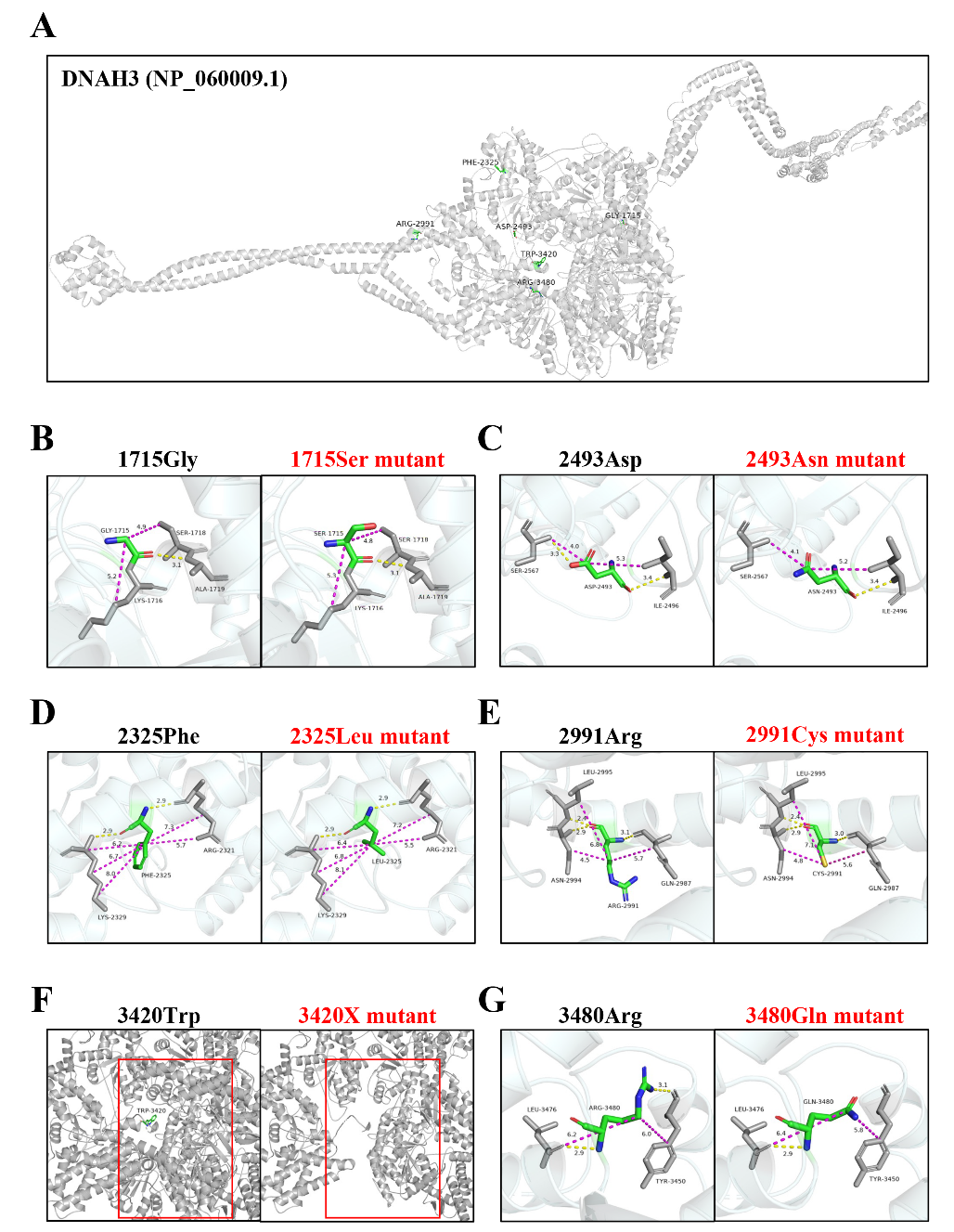


**Supplementary Figure S1. Structural model prediction of the DNAH3 variants**

1. The three-dimensional model of wild-type DNAH3 protein (NP_060009.1).

(B-C) Structural model of the DNAH3 amino acid p.Gly1715Ser substitution and p.Asp2493Asn substitution in P1. The 1715Ser mutant showed altered distance (the purple dash lines) between atoms of Ser1715 and Lys1716/Ser1718. The 2493Asn mutant showed disrupted hydrogen bonding with Ser2567 (the yellow dash line) and altered distance (the purple dash lines) between atoms of Asn2493 and Ser2567/Ile2496.

(D-E) Structural model of the DNAH3 amino acid p.Phe2325Leu substitution and p.Arg2991Cys substitution in P2. The 2325Leu mutant showed altered distance (the purple dash lines) between atoms of Leu2325 and Lys2329/Arg2321. The 2991Cys mutant showed altered distance (the purple dash lines) between atoms of Cys2991and Asn2994/Gln2987 and altered distance of the hydrogen bonding with Gln2981 (the yellow dash line).

(F-G) Structural model of the DNAH3 amino acid p.Trp3420X substitution and p.Arg3480Gln substitution in P3. The 3420X mutant showed broken protein structure (displayed in the red square). The 3480Gln mutant showed disrupted hydrogen bonding with Tyr3450 (the yellow dash line) and altered distance (the purple dash lines) between atoms of Gln3480 and Leu3476/Tyr3450.


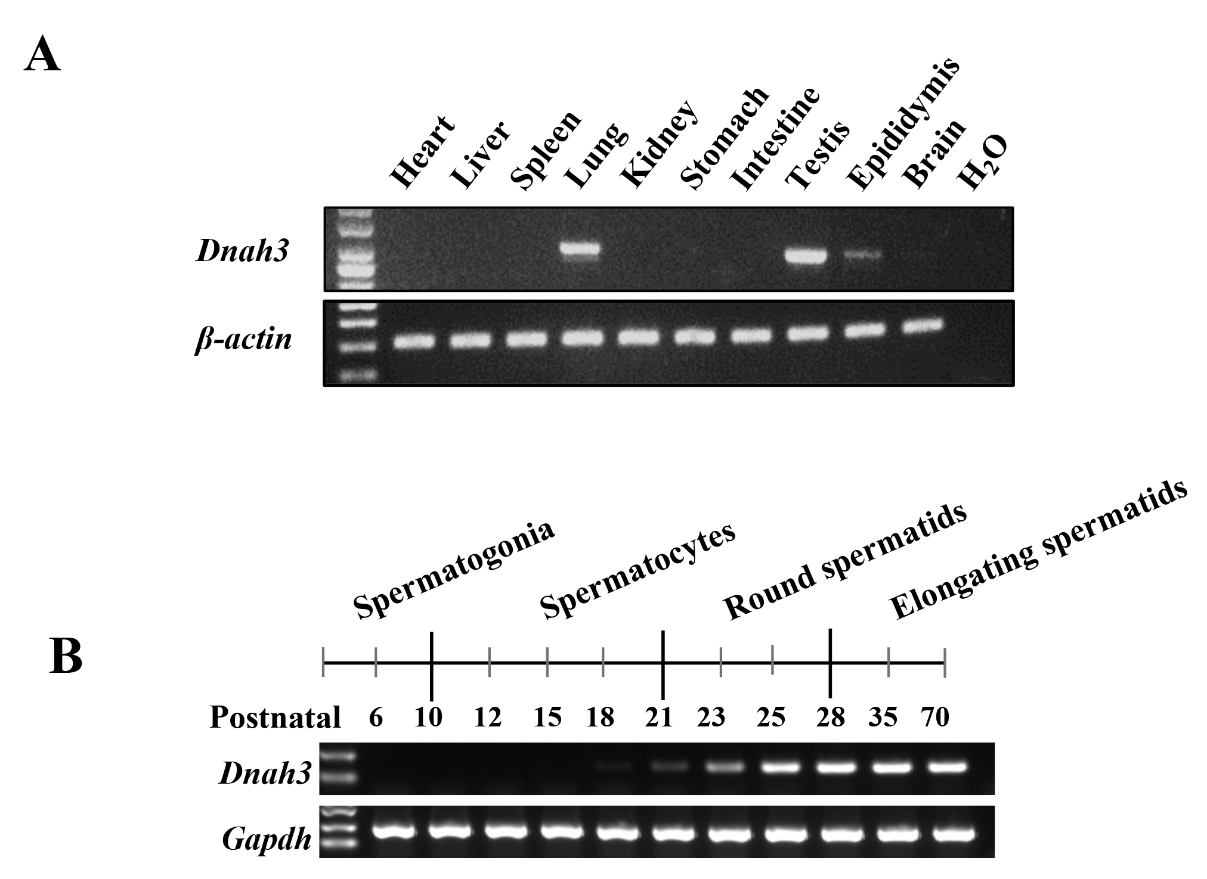


**Supplementary Figure S2. Expression level of *Dnah3* gene in different mouse tissues.**

(A) Expressions of *Dnah3* mRNA in different tissues from adult WT male C57BL/6 mice. The housekeeping gene *β-actin* was used as a control.

(B) Expressions of *Dnah3* mRNA in testes of WT male mice at different ages. The housekeeping gene *Gapdh* was used as a control.

**
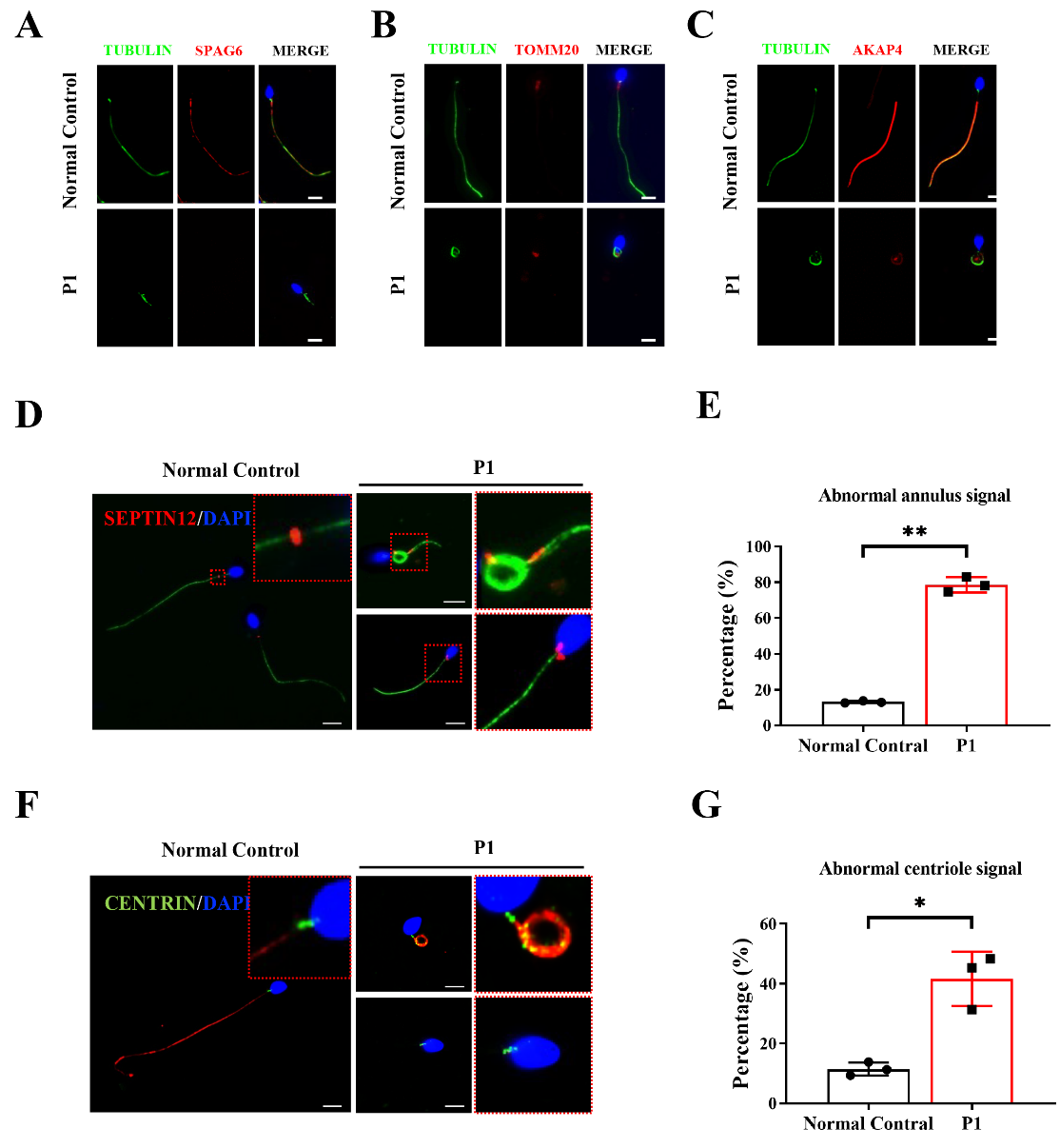
**

**Supplementary Figure S3. Immunofluorescence staining of spermatozoa from the patient from family I.**

(A-C) Spermatozoa were stained with anti-SPAG6 (red), anti-TOMM20 (red), and anti-AKAP4 (red), respectively. Anti-α-TUBULIN (green) and DAPI (blue) were used to mark the sperm flagella and the nuclei of the sperm, respectively. Compared to the normal control, the sperm from patients displayed an absent signal of SPAG6 and mislocated signals of TOMM20 and AKAP4. Scale bar, 5 μm.

(D) Spermatozoa were stained with anti-SEPTIN12 (red). Anti-α-TUBULIN (green) and DAPI (blue) were used to mark the sperm flagella and the nuclei of the sperm, respectively. An intact SEPTIN12 signal was observed in the spermatozoa from normal control. However, the mislocated and disrupted SEPTIN12 signals were observed in the spermatozoa of the patient. Scale bar, 5 μm.

(E) Statistical data showing that the percentage of abnormal SEPTIN12 signal in the sperm from patient was significantly higher than that in the normal control. **p<0.01.

(F) Spermatozoa were stained with anti-CENTRIN (green). Anti-α-TUBULIN (red) and DAPI (blue) were used to mark the sperm flagella and the nuclei of the sperm, respectively. Two adjacent CENTRIN signals were observed in neck of the spermatozoa from the normal control; however, more than two CENTRIN signals were presented along the tail of the spermatozoa from patient. Scale bar, 5 μm.

(G) Statistical data showing that the percentage of abnormal CENTRIN signal in the sperm from patient was significantly higher than that in the normal control. *p<0.05.

**
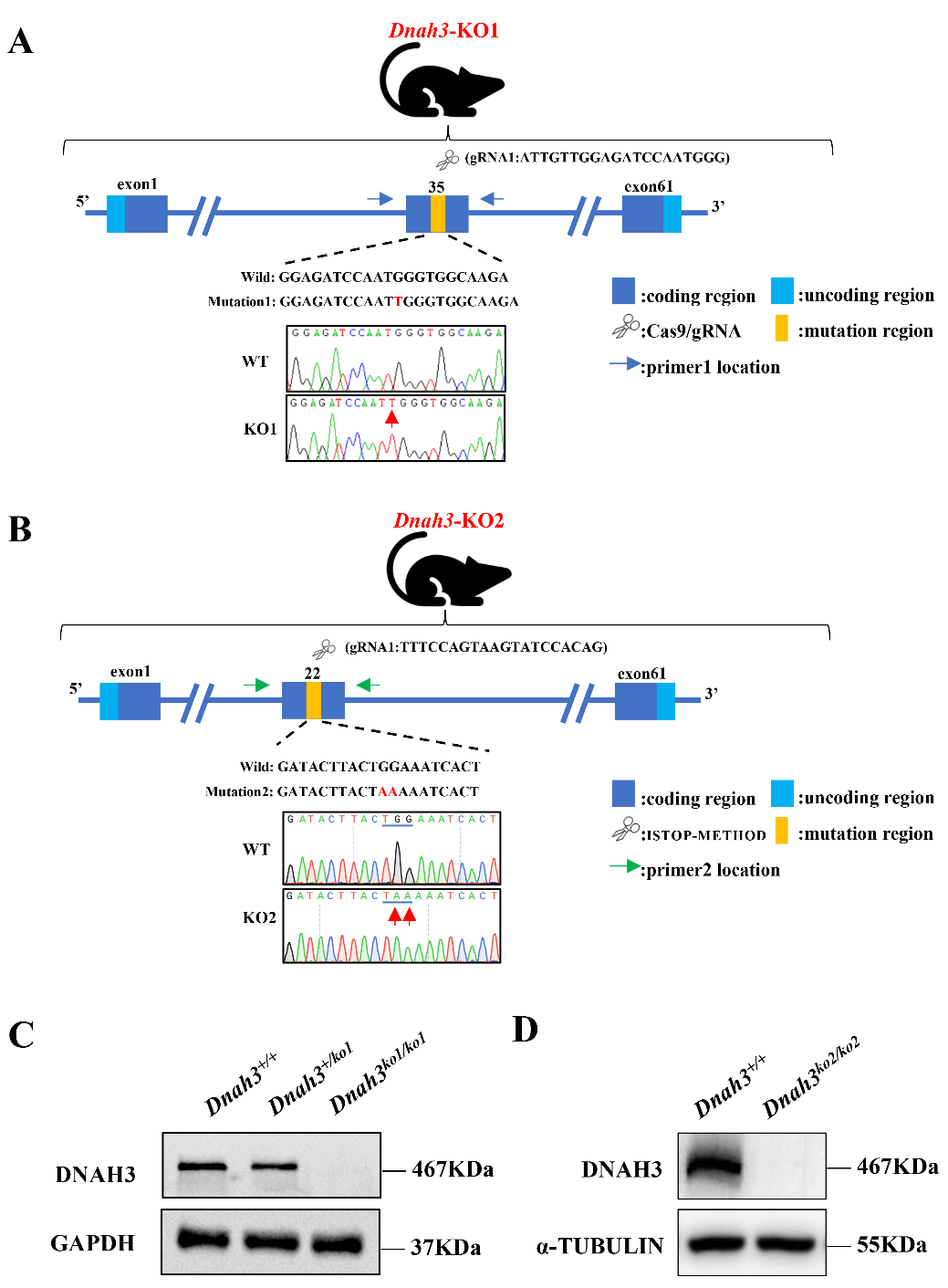
**

**Supplementary Figure S4. Schematic representation of the strategies for generating two *Dnah3* knockout mouse models**

(A) Generation of *Dnah3^ko1/ko1^* mouse model. A frameshift mutation c.5039_5040insT (p.M1680I fs*9) was generated in exon 35 of the mouse *Dnah3* gene using CRISPR/Cas9 technology. The genotypes of wild-type (*Dnah3^+/+^*) and homozygous (*Dnah3^ko1/ko1^*) mice were verified by Sanger sequencing. The red arrowhead represents positions of the mutation.

(B) Generation of *Dnah3^ko2/ko2^* mouse model. A nonsense mutation c.3227_3228GG>AA (p.Trp1076X) was generated in exon 22 of the mouse *Dnah3* gene by inducing a TAA STOP-codon. The genotypes of wild-type (*Dnah3^+/+^*) and homozygous (*Dnah3^ko2/ko2^*) mice were verified by Sanger sequencing. The red arrowhead represents positions of the mutation.

(C) Expression of the DNAH3 protein in the testes of *Dnah3^+/+^*, *Dnah3^+/ko1^*, and *Dnah3^ko1/ko1^* was detected by immunoblotting. GAPDH was used as the loading control.

(D) Expression of the DNAH3 protein in the testes of *Dnah3^+/+^* and *Dnah3^ko2/ko2^* mice was detected by immunoblotting. α-TUBULIN was used as the loading control.


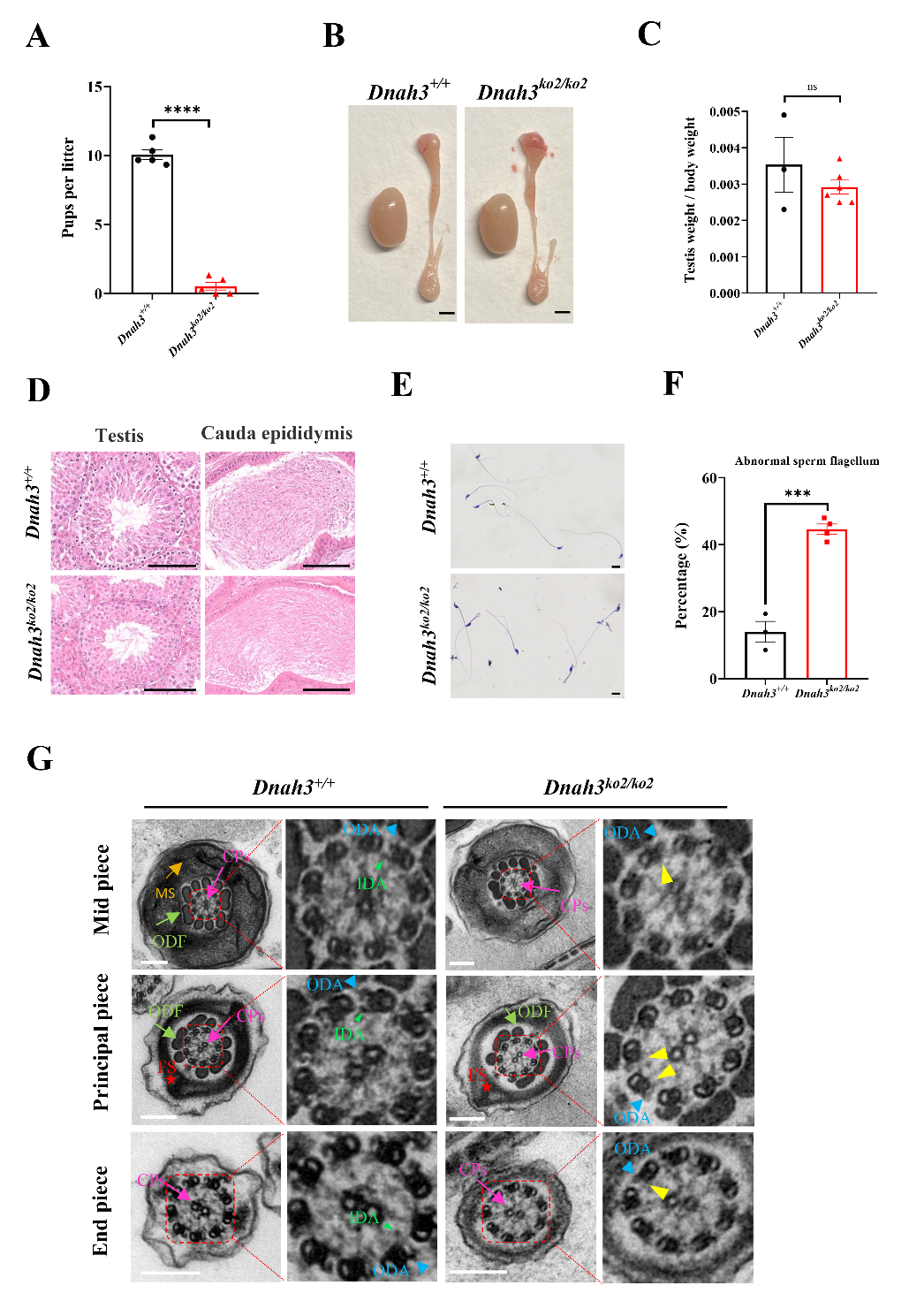


**Supplementary Figure S5. *Dnah3^ko2/ko2^* male mice displayed severely damaged male fertility and asthenoteratozoospermia.**

(A) Fertility test of *Dnah3^+/+^* and *Dnah3^ko2/ko2^* male mice at 11 weeks of age. Two *Dnah3^ko2/ko2^* male mice showed complete sterility, while the other three male mice showed subfertility. ****p<0.0001, n=5.

(B) Gross morphology of the testis and epididymis of *Dnah3^+/+^* and *Dnah3^ko2/ko2^* male mice. Scale bar, 2 mm.

(C) Analysis of the testis weight/body weight ratios between *Dnah3^+/+^* and *Dnah3^ko2/ko2^* male mice at 10 weeks of age. ns indicates not significant.

(D) H&E staining of seminiferous tubules and cauda epididymis from 10-week-old *Dnah3^+/+^* and *Dnah3^ko2/ko2^* male mice. Scale bars, 100 μm.

(E) H&E staining of epididymis spermatozoa from 10-week-old *Dnah3^+/+^* and *Dnah3^ko2/ko2^* male mice. An aberrant curved flagellar morphology was found in spermatozoa of *Dnah3^ko2/ko2^* male mice. Scale bars, 10 μm.

(F) Statistical data showing that the percentage of abnormal sperm flagellum in *Dnah3^ko2/ko2^* male mice was significantly higher than that in *Dnah3^+/+^* male mice. ***p<0.001.

(G) Transmission electron microscopy analysis of cross-sectional ultrastructure of cauda epididymal spermatozoa from *Dnah3^+/+^* and *Dnah3^ko2/ko2^* male mice at 10 weeks of age. The typical "9+2" axoneme structure and axonemal accessory structure, including central pairs (CPs; pink arrow), mitochondrial sheath (MS; yellow arrow), outer dense fibres (ODFs; green arrow), fibrous sheath (FS; red asterisk), and outer dynein arms (ODAs; blue triangle) were visible and intact in the spermatozoa from both *Dnah3^+/+^* and *Dnah3^ko2/ko2^* male mice. However, partial loss of inner dynein arms (indicated by yellow triangles) happened in *Dnah3^ko2/ko2^* male mice compared with the intact IDAs (green triangle) in the *Dnah3^+/+^* male mice. The structures in the red dotted square are magnified proportionally on the right side. Scale bars, 200 nm.

**
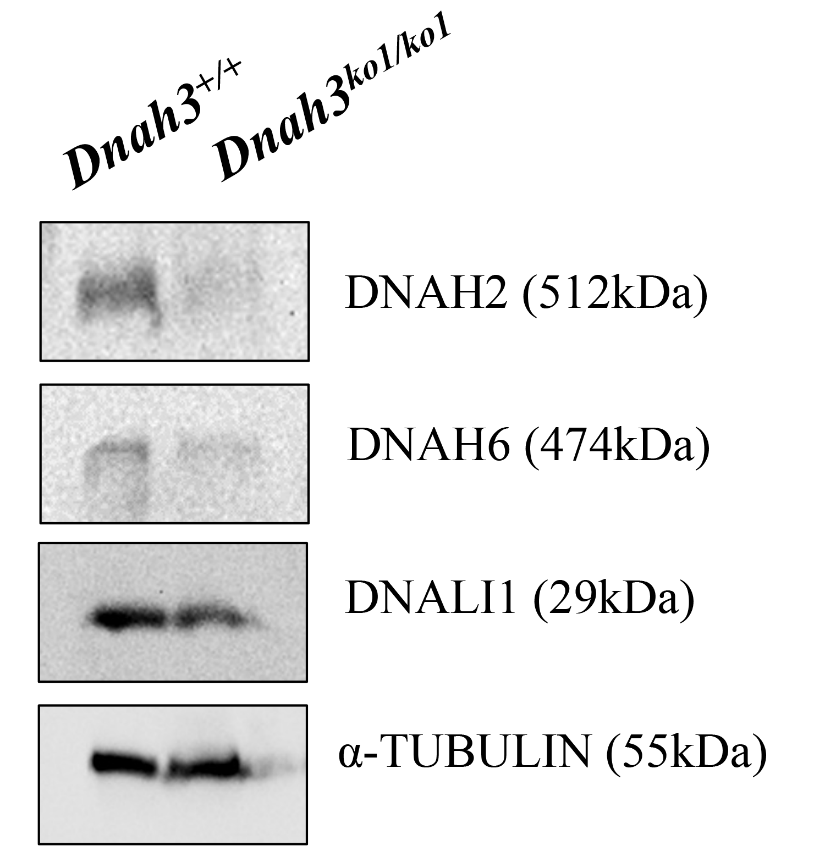
**

**Supplementary Figure S6. Expression of various IDA-associated proteins in the testis from *Dnah3^ko1/ko1^* male mice.**

The protein expressions of DNAH2, DNAH6, and DNALI1 in the testis of *Dnah3^ko1/ko1^* male mice were found to be reduced compared to that of *Dnah3^+/+^* male mice through immunoblotting analysis. α-TUBULIN was used as the loading control.


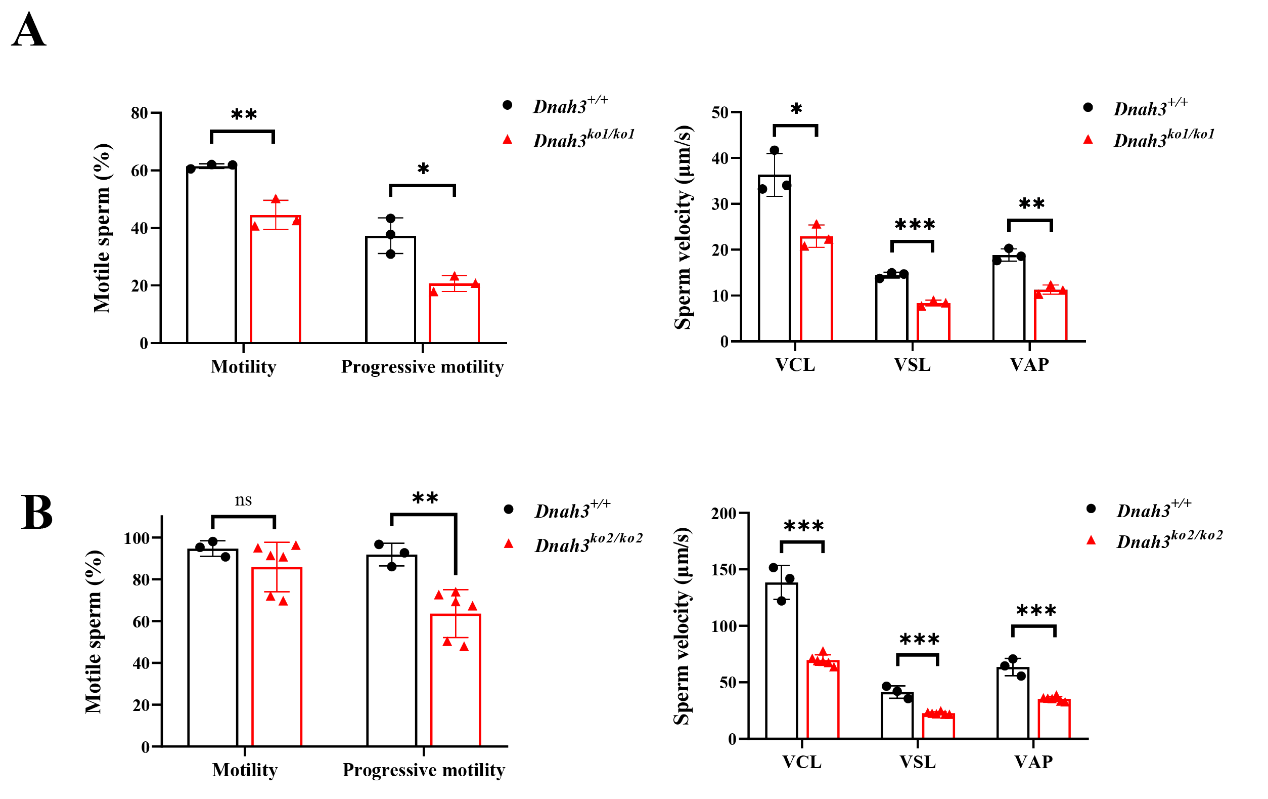


**Supplementary Figure S7. Computer-aided sperm analysis of the sperm motility and velocity in *Dnah3^ko1/ko1^* and *Dnah3^ko2/ko2^* male mice.**

Both *Dnah3^ko1/ko1^* male mice (A) and *Dnah3^ko2/ko2^* male mice (B) showed reduced sperm motility, progressive motility, and reduced sperm velocity parameters, such as curvilinear velocity (VCL), straight-line velocity (VSL), and average path velocity (VAP).


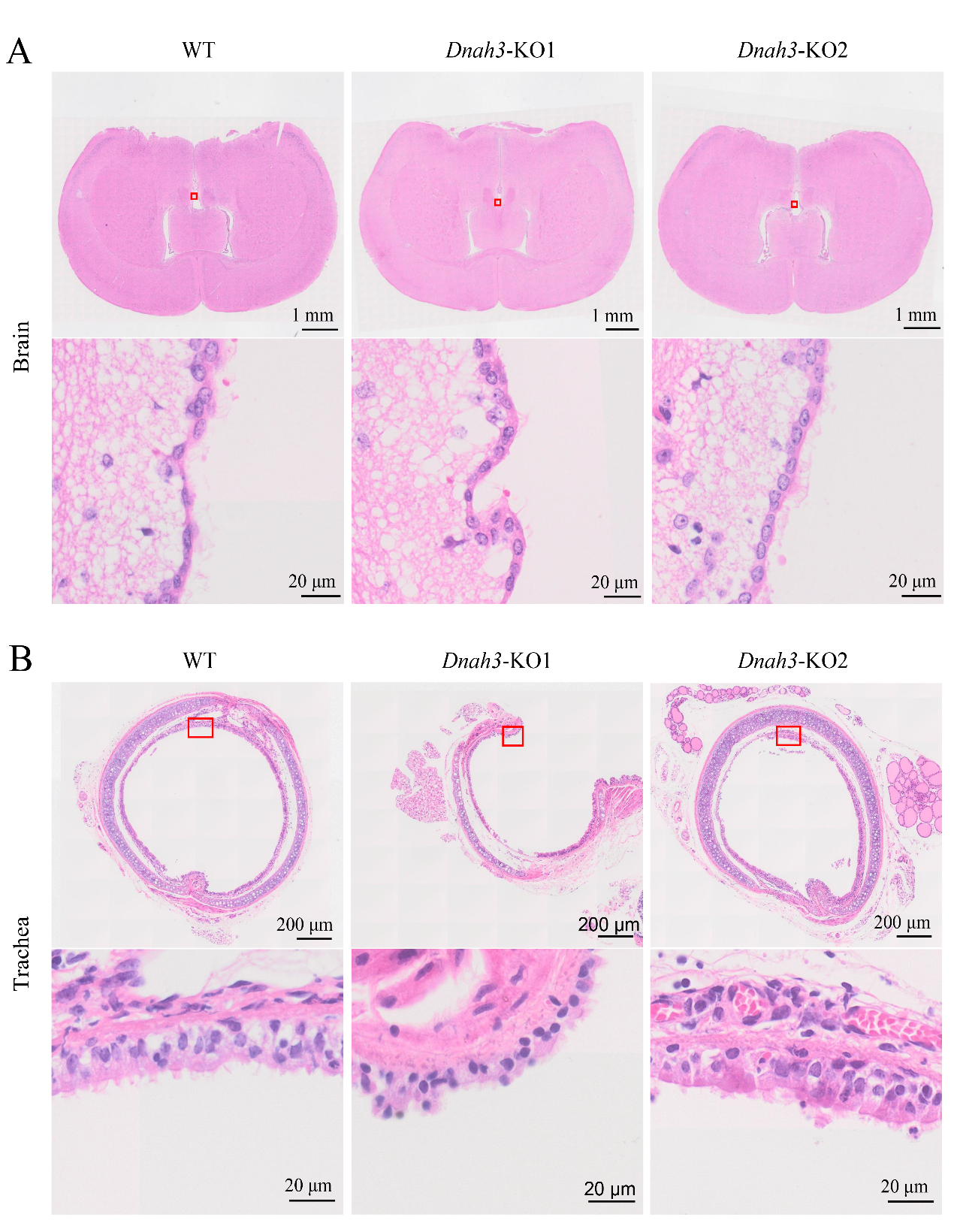


**Supplementary Figure S8. H&E staining of brain and trachea of adult WT and *Dnah3*-KO male mice.**

H&E staining of the coronal brain (A) and trachea (B) cross-sections of adult WT and *Dnah3*-KO male mice, respectively. The cilia from trachea or brain were not impaired in Dnah3-KO mice compared with WT controls. The high magnifications of the boxed areas are shown below.
