# Supplementary material for: Bi-allelic variants in DNAH3 cause male infertility with asthenoteratozoospermia in humans and mice
Source: Hum Reprod Open. 2024 Jan 11;2024(1):hoae003. doi: 10.1093/hropen/hoae003 (PMC10834362; doi:10.1093/hropen/hoae003)
Supplement: hoae003_Supplementary_Data [file hoae003_supplementary_data.zip › HRO-23-0175-R2-SuppTables1-5.docx]

**Supplementary Table S1. Primers used for verification of the *DNAH3* variants identified in probands.**

| **Primer ID** | **Sequence (5′ to 3′)** |
| --- | --- |
| DNAH3-E36-F: | GAACCGAAGTTCTCCAGAGGT |
| DNAH3-E36-R: | CGATCGAGGCCTGACAAGAC |
| DNAH3-E47-F: | CTGCAGGTCAGCCACTATCA |
| DNAH3-E47-R: | CAACAGGAAGGGAACCTGAA |
| DNAH3-E48-F: | TAGGATCTGCCGTGTCCTGA |
| DNAH3-E48-R: | ATGTCAGCCTTCTCGTCAGC |
| DNAH3-E53-1F: | CAGATGCAGAAGCTGAACCA |
| DNAH3-E53-1R: | AGTCACTGAAGCCAGGGATG |
| DNAH3-E53-2F: | CTCTCTCCTCCTGACCATCG |
| DNAH3-E53-2R: | GCCTTCAAAGCCATCTGAAC |

**Supplementary Table S2. Primers used for genotyping of the *Dnah3* knockout mouse.**

| **Primer ID** | **Sequence (5′ to 3′)** |
| --- | --- |
| Dnah3-ko1-F | AGGGCAGTGCACCAGAATTA |
| Dnah3-ko1-R | ACCTCCCTAAAGCAGAGGGT |
| Dnah3-ko2-F | CTGAAGACTGGCAGGTTACTG |
| Dnah3-ko2-R | CTGTTCTCCTTTCCATATCCC |

**Supplementary Table S3. Primers used for reverse-transcription PCR**

| **Primer ID** | **Sequence (5′ to 3′)** |
| --- | --- |
| Dnah3-F1 | AGTGTTCTGTTGCTCCGGG |
| Dnah3-R1 | AAAGCAGCCATACAGTTGCC |
| Dnah3-F2 | GCACACAGAACTGGTTGAGGA |
| Dnah3-R2 | TCCAGCAGGGAGGAGTACAG |
| β-actin-F | CCTGGCACCCAGCACAAT |
| β-actin-R | GGGCCGGACTCGTCATAC |
| Gapdh-F | GTCGTGGAGTCTACTGGTGTC |
| Gapdh-R | GAGCCCTTCCACAATGCCAAA |

| **Supplementary Table S4. Detailed description of the biallelic variants in *DNAH3* identified in three infertile men** | | | | | | | | | | | | | | |
| --- | --- | --- | --- | --- | --- | --- | --- | --- | --- | --- | --- | --- | --- | --- |
| **Individual** | **Gene** | **Location [Hg19]** | **Exon** | **cDNA Alteration** | **Amino Acid Alteration** | **Mutation Zygosity** | **Function** | **Allele Frequency** | | |  | ***In-silico* bioinformatics prediction** | | |
|  |  |  |  |  |  |  |  | **1000 Genomes** | **gnomAD** | **gnomAD-EAS** |  | **PolyPhen-2** | **Mutation Taster** | **CADD** |
|  |  |  |  |  |  |  |  |  |  |  |  |  |  |  |
| P1 | *DNAH3* | chr16:21045350 | 36 | c.G5143A | p.Gly1715Ser | HET | missense | NA | NA | NA |  | PD | DC | 27 |
|  |  | chr16:20996587 | 48 | c.G7477A | p.Asp2493Asn | HET | missense | NA | NA | NA |  | PD | DC | 27.9 |
| P2 | *DNAH3* | chr16:20998680 | 47 | c.T6973C | p.Phe2325Leu | HET | missense | NA | 0.00003229 | 0.0006 |  | PD | DC | 28.1 |
|  |  | chr16:20976235 | 53 | c.C8971T | p.Arg2991Cys | HET | missense | NA | 0.0000323 | 0 |  | PD | Polymorphism | 21.1 |
| P3 | *DNAH3* | chr16:20974946 | 53 | c.G10260A | p.Trp3420X | HET | nonsense | NA | NA | NA |  | NA | DC | 43 |
|  |  | chr16:20974767 | 53 | c.G10439A | p.Arg3480Gln | HET | missense | NA | 0.0002 | 0 |  | PD | DC | 29.9 |

**Note**:

1. The reference transcript of *DNAH3* is NM_017539.2, NP_060009.1.

2. HOM: homozygous, HET: heterozygous.

3. PloyPhen-2 score: ranges from 0.000 to 1.000, and 0.000 is benign and 1.000 is damaging, PD: possible damaging.

4. MutationTaster: the probability value refers to the prediction, i.e. a value close to 1 indicates a high 'security' of the prediction, DC: disease causing.

5. CADD score: amino acid substitution is predicted damaging if the score is >4.

6. NA: not available.

Web resources:

1000 Genomes Project, https://www.internationalgenome.org/

gnomAD, https://gnomad.broadinstitute.org

PolyPhen-2, http://genetics.bwh.harvard.edu/pph2/

Mutation Taster, https://www.mutationtaster.org/

CADD, <https://cadd.gs.washington.edu/snv>

**Supplementary Table S5 Analysis of potential off-target mutations**

| **Targeted sites** | **Potential**  **off-target sites** | **Sequence** | **Mismatches** | **Analyzed**  **mice** | **Genotype** |
| --- | --- | --- | --- | --- | --- |
| KO1 mouse  *Dnah3* sgRNA1:  chr7: 120016492  ATTGTTGGAGATCCAATGGGTGG | chr5:64609766 | ATTGTgGGAGATgCAAaGGGAGG | 3 | KO1 #1-#4 | WT |
|  | chr5:97102744 | AcTGTTGGAGcTCCcATGGGAGG | 3 | KO1 #1-#4 | WT |
|  | chr1:46207615 | ATTGTTGGAGAaCCAtTtGGAGG | 3 | KO1 #1-#4 | WT |
|  | chr1:46632270 | ATTGTTGGAGAgCCAtTtGGAGG | 3 | KO1 #1-#4 | WT |
|  | chr1:53540933 | ATTGTTGGAGAgCCAtTtGGAGG | 3 | KO1 #1-#4 | WT |
|  | chr7:17098113 | ATTGTTGaAGAgCCAATGGaTGG | 3 | KO1 #1-#4 | WT |
|  | chr14:101360134 | ATTGcTGaAGAaCCAATGGGAGG | 3 | KO1 #1-#4 | WT |
|  | chr6:103779816 | ATTGTTGGAGtaCCAtTGGGAGG | 3 | KO1 #1-#4 | WT |
|  | chr11:68264346 | tTTGTTGGgGcTCCAATGGGGGG | 3 | KO1 #1-#4 | WT |
|  | chr13:75353267 | ATTGcTGGAcATCCAATGGcTGG | 3 | KO1 #1-#4 | WT |
|  | chr18:34189256 | ATTGgTtGAtATCCAATGGGAGG | 3 | KO1 #1-#4 | WT |
|  | chr18:39855559 | ATTcTTGGAGATCCAgTaGGAGG | 3 | KO1 #1-#4 | WT |
|  | chr18:90550647 | ATTtTTGGAGATaCAAaGGGAGG | 3 | KO1 #1-#4 | WT |
|  | chr3:67149179 | AcaGTTGGAGATCCAgTGGGAGG | 3 | KO1 #1-#4 | WT |
|  | chr3:90548966 | ATTGTTGtAcATCCAATGGcTGG | 3 | KO1 #1-#4 | WT |
| KO2 mouse  *Dnah3* sgRNA2:  chr7: 120047540  TTTCCAGTAAGTATCCACAGTGG | chr8:35972213 | TaTCCAGTAAGTATCCttAGAGG | 3 | KO2 #1-#4 | WT |
|  | chr15:99924156 | TTTCCAGaAAGaATCCACtGAGG | 3 | KO2 #1-#4 | WT |
|  | chr1:51268908 | TTTCaAGTAAGTAgCCtCAGAGG | 3 | KO2 #1-#4 | WT |
|  | chr7:118720016 | TTTCCAGctgGTATCCACAGAGG | 3 | KO2 #1-#4 | WT |
|  | chr7:132157045 | TTTCaAGTgAGTATCCcCAGAGG | 3 | KO2 #1-#4 | WT |
|  | chr2:54512119 | TTTCaAGcAAGcATCCACAGAGG | 3 | KO2 #1-#4 | WT |
|  | chr2:140428872 | TTTtCAGTAAtTATtCACAGTGG | 3 | KO2 #1-#4 | WT |
|  | chr12:49652981 | TTTCCAtgAtGTATCCACAGAGG | 3 | KO2 #1-#4 | WT |
|  | chr4:8561477 | TTTCCAGTAAGcAcCCAaAGTGG | 3 | KO2 #1-#4 | WT |
|  | chr4:33063399 | TTTCCAGTAgGTtTCCcCAGAGG | 3 | KO2 #1-#4 | WT |
|  | chr16:85050017 | TTctCAGTAAtTATCCACAGAGG | 3 | KO2 #1-#4 | WT |
|  | chr16:95781855 | TTTCCAGTgAGTAaCCAaAGGGG | 3 | KO2 #1-#4 | WT |
|  | chr9:113232196 | TTcCCAGcAtGTATCCACAGAGG | 3 | KO2 #1-#4 | WT |
|  | chrX:147432733 | TTTCCAcTtAGTATgCACAGTGG | 3 | KO2 #1-#4 | WT |
|  | chr6:99167642 | TTTCCAGaAgGTtTCCACAGAGG | 3 | KO2 #1-#4 | WT |
|  | chr13:67410110 | TTTaaAGTAAGTATCCACAaAGG | 3 | KO2 #1-#4 | WT |
|  | chr3:60139103 | TTTCCAtTAAGgATCCACAGTGG | 2 | KO2 #1-#4 | WT |
